# Supplementary material for: The SecA motor generates mechanical force during protein translocation
Source: Nat Commun. 2020 Jul 30;11:3802. doi: 10.1038/s41467-020-17561-2 (PMC7393111; doi:10.1038/s41467-020-17561-2)
Supplement: Supplementary file 1 — Supplementary Information [file 41467_2020_17561_MOESM1_ESM.pdf]

Supplementary Information

The SecA motor generates mechanical force to unfold translocating proteins

Riti Gupta, Dmitri Toptygin and Christian M. Kaiser

Supplementary information contains

- Supplementary Figures 1 to 5
- Supplementary Tables 1 and 2
- Supplementary Methods
- Supplementary References

Supplementary Figures

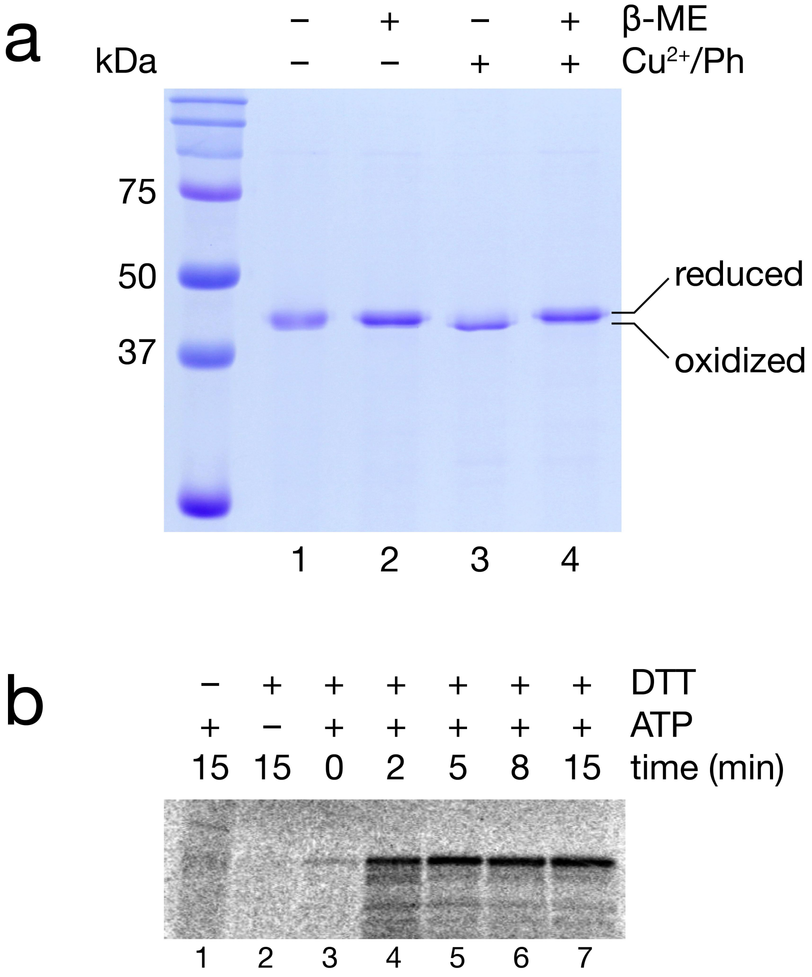

Supplementary Figure 1. Disulfide loop formation reversibly stalls translocation. a. Loop formation of pOA-mDHFR. pOA-mDHFR was incubated with copper/phenanthroline (Cu^{2+}/Ph) to catalyze the formation of an intramolecular disulfide bond. Oxidized pOA-mDHFR migrates with higher electrophoretic mobility than the reduced form, which is expected for a polypeptide containing a disulfide loop (lane 3 vs. lane 4). **b.** Translocation of the pOA-mDHFR substrate protein assessed by protease protection. Radiolabeled oxidized protein was added to SecYEG/SecA proteoliposomes and incubated at 37°C for the time indicated. The sample was then subjected to proteinase K digestion, and products were analyzed by SDS-PAGE and autoradiography. In the absence of DTT or ATP, little protected substrate protein is observed (lanes 1 and 2). When both ATP and DTT are added, an increasing amount of protected protein is observed (lanes 3 to 7), indicating translocation into the interior of the proteoliposomes.

Source data are provided as a Source Data file.

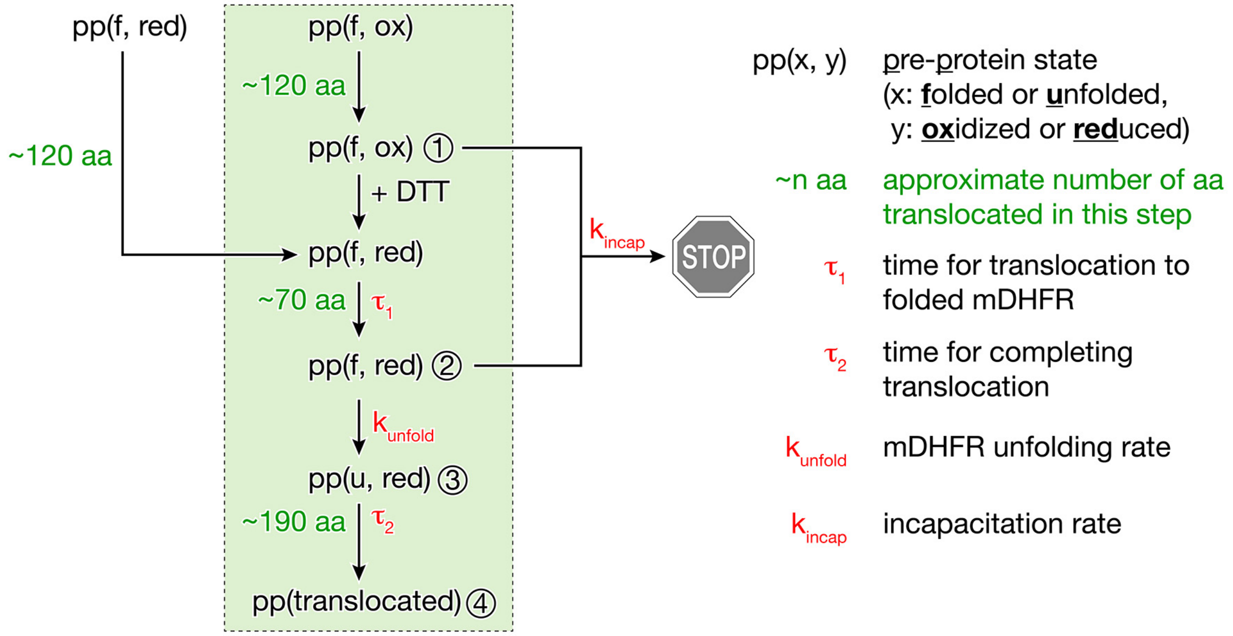

Supplementary Figure 2. Schematic of our kinetic model of translocation. The luminescence signal readings from translocation experiments reflect the kinetics of multiple processes. The processes relevant to our analyses are shown in the green box. At the beginning of the measurement, the preprotein (pp) is primarily in an oxidized and folded state (pp(f,ox)). In the presence of ATP, translocation of the first ~120 amino acids proceeds until stalling at the disulfide loop (①). DTT is added after a waiting time that allows accumulation of stalled substrate protein, yielding reduced preprotein (pp(f,red)). Translocation of the next ~70 amino acids proceeds with the time constant τ_1 until the translocon encounters folded mDHFR (②). After unfolding of mDHFR with the rate k_{unfold}, the unfolded preprotein (pp(u, red)) (③) completes translocation with the time constant τ_2, resulting in translocated preprotein (pp(translocated)) inside the proteoliposomes (④). The model accounts for the presence of reduced preprotein that undergoes translocation independently of DTT addition (left). An irreversible side process that we term incapacitation (right) occurs with rate k_{incap}, resulting in reduced amounts of translocated protein. Additional parameters in the model include the depletion of the luciferase small molecule substrate and instrument sensitivity, which are not shown in this diagram. The fit parameters that yield the translocation and unfolding rates are shown in red here. See Supplementary Information for a detailed description of the model.

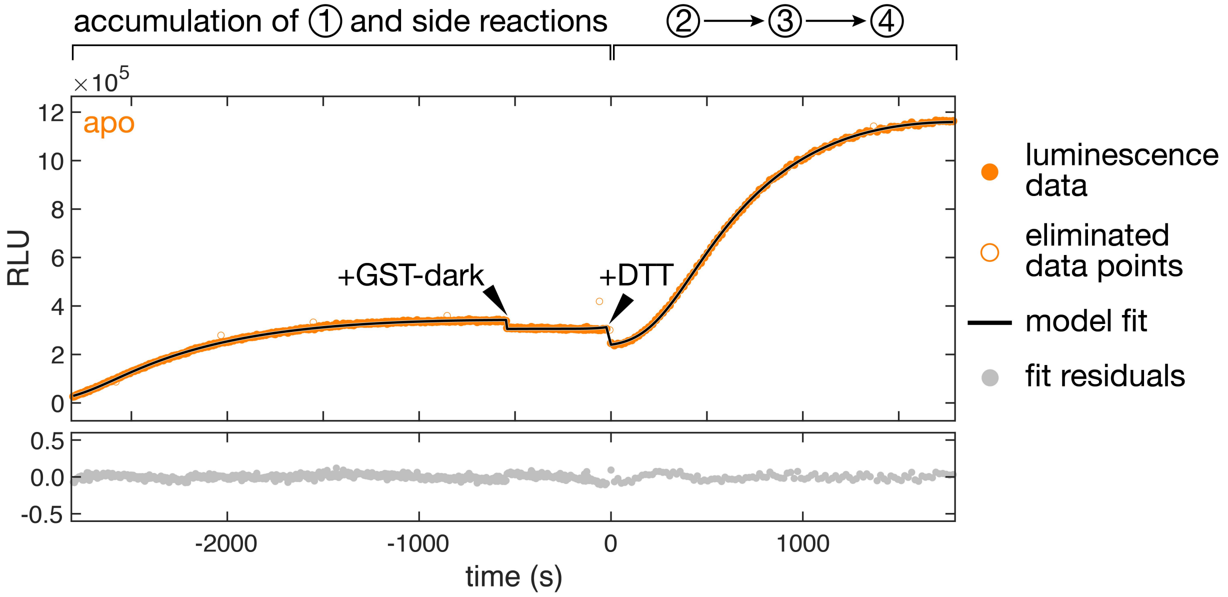

Supplementary Figure 3. Real-time translocation measurement. Complete luminescence recording (upper graph) for a translocation experiment with pOA-mDHFR in the absence of ligands starting after mixing SecYEG/SecA proteoliposomes containing encapsulated 11S NanoLuc, ATP, GST-dark and oxidized pOA-mDHFR substrate protein. Closed orange circles represent the data used to calculate a fit (black line) based on our model. Open circles represent data points that were eliminated from analysis because they had an unusually high variance (9 out of 566 samples in this recording; see Supplementary Information, section 1, for details). The initial increase results from side reactions (presumably import of non-oxidized substrate protein molecules) that occur while stalled translocation substrate accumulates. Arrowheads indicate the addition of extra GST-dark and DTT to the reaction. Additional GST-dark quenches any 11S protein that is accessible on the outside of the proteoliposomes. DTT reduces the substrate protein, restarting translocation. The reaction stages are indicated on top, using the same numbers as in Figure 1c. The graph on the bottom shows the residuals from the fit.

Source data are provided as a Source Data file.

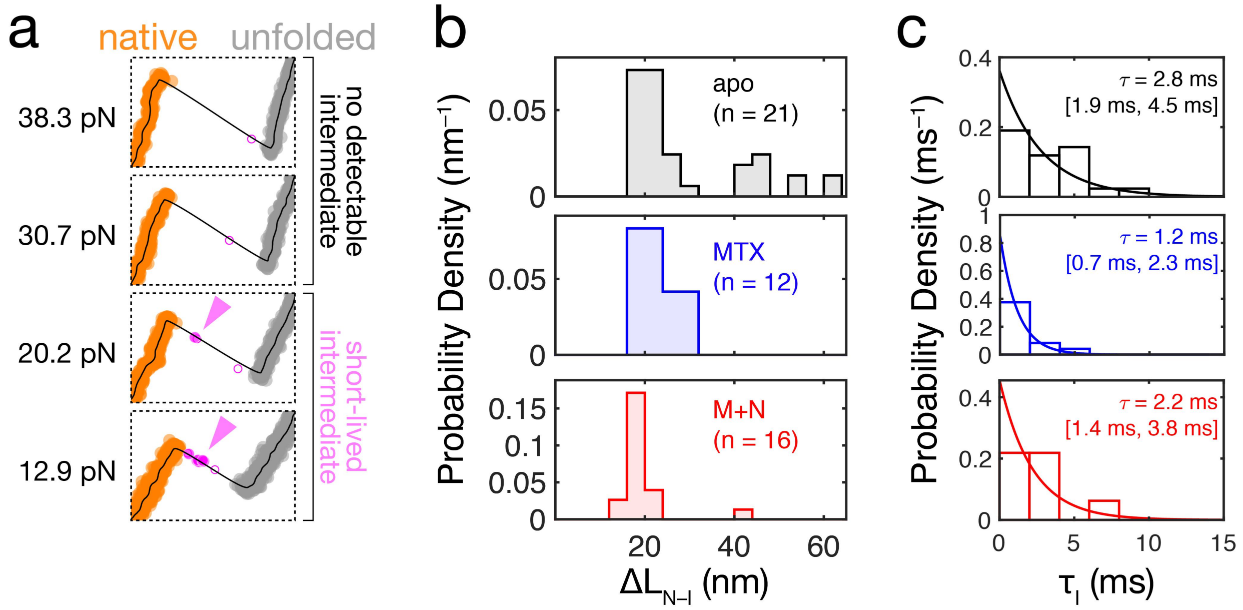

Supplementary Figure 4. mDHFR populates a transient unfolding intermediate. **a.** Blow-ups of the region around the unfolding transition for four apo-mDHFR molecules. Raw data is shown as circles, colored by state (orange: folded, grey: unfolded), with samples that fall between the two states in magenta. One or two samples between folded and unfolded can result from time-averaging (white with magenta outline). The majority of traces do not exhibit a detectable intermediate (top two examples). Additional samples in the transition region (solid magenta) represent a transient unfolding intermediate, which is apparent in some traces (bottom two examples, arrowheads). **b.** Histograms of the contour length changes for native-to-intermediate transitions for the three ligand conditions (apo, MTX, M+N). Most transitions result in a contour length change of ~20 nm. The number of traces with a detected unfolding intermediate is indicated in parentheses. **c.** Histograms of intermediate lifetimes (bars) with fit to an exponential probability density function (line). Almost all detected intermediates unfold within 10 ms. The fits yield mean lifetimes of approximately 2 ms. However, our time resolution of 1 ms results in many missed events, and the true intermediate lifetime may be smaller than this value. The lifetimes do not appear to increase in the presence of ligands.

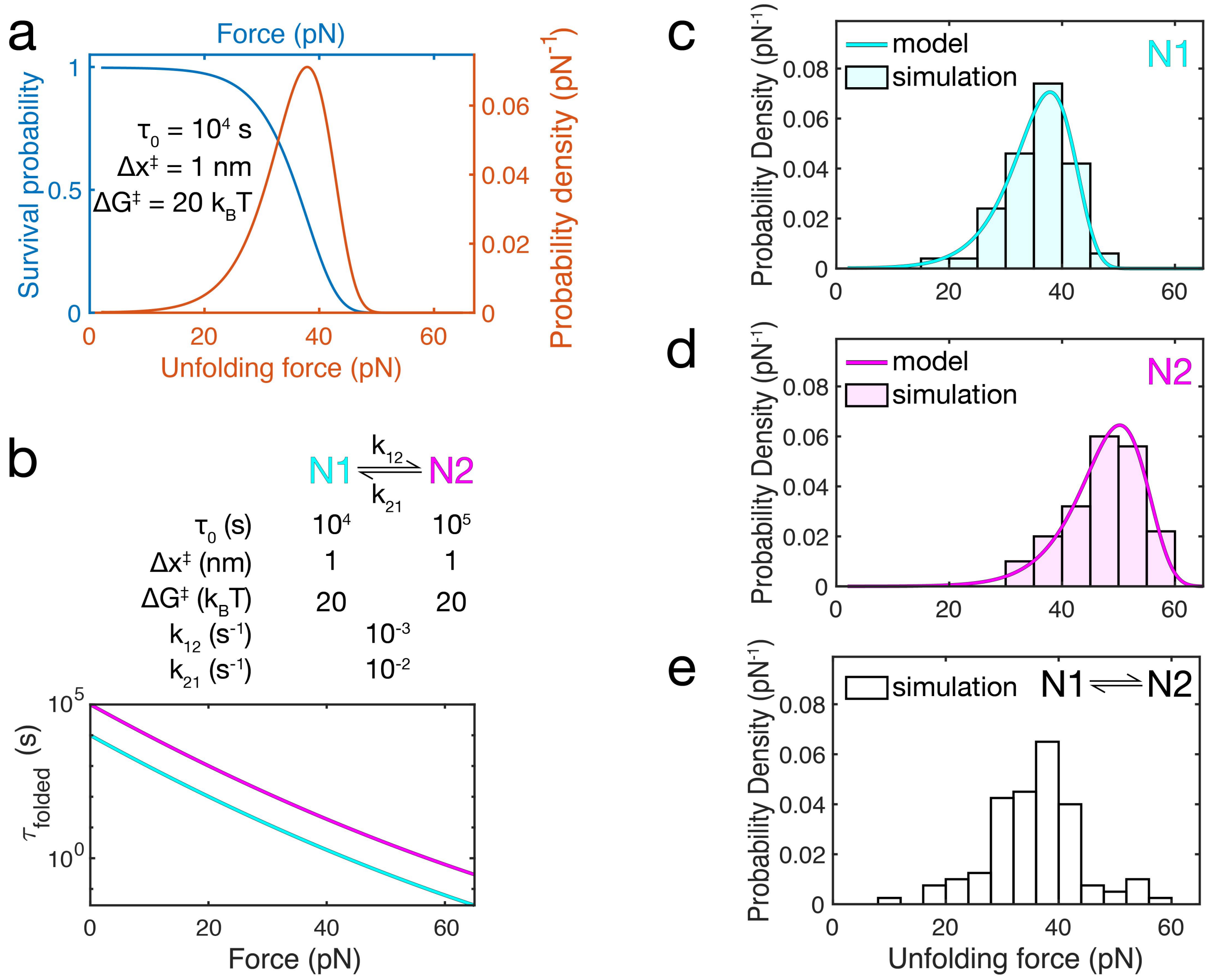

Supplementary Figure 5. Illustration of state switching effects on unfolding forces. **a.** Calculated survival probability of the folded state (blue) and unfolding force probability density (orange) in a force ramp experiment using the parameters indicated in the figure. The probability of the folded state staying intact decreases as the force is ramped up (blue line). As a result, the probability density of the observed unfolding forces (orange line) shows a skewed distribution that drops sharply at high forces. **b.** Parameters for a state switching model and calculated force-dependent lifetimes. In the hypothetical model, two states (N1 and N2) that differ in their intrinsic folded state lifetimes interconvert with constant rates (k_{12} and k_{21}). **c, d.** Calculated (lines) and simulated (bars) lifetimes distributions for states N1 and N2. The simulated data (100 events) shows the expected distributions. **e.** Simulated data for a state-switching model. The simulated unfolding forces (100 events) show a broad distribution with a tail at high unfolding forces, similar to the experimentally observed distribution for mDHFR (Figure 2f). The parameters for simulating N1 and N2 unfolding were chosen to illustrate how state-switching manifests in unfolding force distributions. They do not reflect the true parameters of apo-mDHFR unfolding, which cannot be reliably determined from the experimental data described in this study.

Source data are provided as a Source Data file.

Supplementary Tables

	apo		MTX		MTX+NADPH	
Parameter	mean	sd	mean	sd	mean	sd
α_1	$4.18\cdot10^{-4}$ s^{-1}	$2.89\cdot10^{-5}$ s^{-1}	$1.92\cdot10^{-4}$ s^{-1}	$8.34\cdot10^{-6}$ s^{-1}	$2.16\cdot10^{-5}$ s^{-1}	$3.23\cdot10^{-6}$ s^{-1}
α_2	$1.10\cdot10^{-3}$ s^{-1}	$1.65\cdot10^{-4}$ s^{-1}	$1.22\cdot10^{-3}$ s^{-1}	$1.48\cdot10^{-4}$ s^{-1}	$9.12\cdot10^{-4}$ s^{-1}	$8.20\cdot10^{-5}$ s^{-1}
α_3	0.986	$2.33\cdot10^{-3}$	0.982	$1.21\cdot10^{-3}$	0.897	$2.61\cdot10^{-2}$
α_4	245 s	6.83 s	250 s	6.92 s	261 s	13.6 s
α_5	148 s	4.24 s	156 s	9.95 s	156 s	13.5 s
α_6	16.0 s	15.9 s	75.9 s	19.5 s	116 s	30.6 s
α_7	131 s	16.0 s	130 s	29.4 s	297 s	36.1 s
α_8	$6.28\cdot10^{-11}$	$1.23\cdot10^{-11}$	$6.28\cdot10^{-11}$	$1.23\cdot10^{-11}$	$6.28\cdot10^{-11}$	$1.23\cdot10^{-11}$
α_9	$6.37\cdot10^{+7}$	$1.69\cdot10^{+7}$	$6.37\cdot10^{+7}$	$1.69\cdot10^{+7}$	$6.37\cdot10^{+7}$	$1.69\cdot10^{+7}$
α_{10}	0.910	0.0239	0.921	0.0304	0.909	0.0479
α_{11}	0.693	0.0156	0.703	0.0196	0.920	0.0597

Supplementary Table 1. Full parameter set for fits to real-time translocation measurements. See Supplementary Information, section 1, Kinetic Model for Fitting Luminescence Data, for a detailed description of the model, and section 2, Numerical Methods for Fitting Luminescence Data, for calculation of means and standard deviations from independent replicates of the ensemble luminescence measurements.

Target	Direction	Sequence
mDHFR	fwd	ggatccgcggccgcgatggttcgaccattgaac
	rev	agccaccacttgatatcgtgtctttcttctcgtagacttcaaac
SecA	fwd	atgctaatcaaattgttaactaaagttttcggtagtcgtaac
	rev	cagtgccatggccgcctgcaataaggatccac
pOA (pOA-mDHFR)	fwd	taaagcttgatccggctgctaacaaagcc
	rev	atcaatttctgggaaaaac
mDHFR (pOA-mDHFR)	fwd	cgtttttcccagaaattgatttggggaaatataaacttctc
	rev	gccggatcaagctttagctgatcttcttgaacagac
11S	fwd	gaaaatcgaatggcacgaaactagtggtggcggtatgg
	rev	atccgccaaaacagccaagggatccgttattaagagttgattgttacacg
GST-dark	fwd	gactccttaagattcttgtacgacggtattagaattc
	rev	gtatcaggctgaaaatcttctctcatccg

Supplementary Table 2. Sequences of primers used for cloning expression constructs.

Supplementary Methods

1. Kinetic Model for Fitting Luminescence Data

In our translocation measurements, the luminescence signal is generated when a p86-tagged substrate protein is fully translocated into the interior of SecYEG/SecA proteoliposomes and restores luciferase activity by binding to 11S. We developed a kinetic model that takes into account the following processes:

(1) The oxidized substrate protein engages with the translocon. After DTT-induced disulfide loop opening, unfolding of mDHFR and completion of translocation, light is generated by the encapsulated NanoLuc luciferase.

(2) Substrate protein with reduced cysteines bypasses disulfide-loop stalling and reaches the interior of the proteoliposomes, generating signal.

(3) Irreversible inactivation (incapacitation) prevents substrate import.

(4) NanoLuc substrate depletion results in a time-dependent decrease of light intensity.

Our model describes the time evolution of the luminescence intensity, I, as a function

$$I = I(t, \boldsymbol{\alpha}) \tag{1}$$

where t is time (the independent variable) and $\boldsymbol{\alpha}$ is a parameter vector with the following components:

- α_1: unfolding rate of mDHFR
- α_2: incapacitation rate
- α_3: molar fraction of preprotein with oxidized disulfide at $t = T_0$
- α_4: mean translocation time for oxidized mDHFR
- α_5: RMS deviation translocation time for oxidized mDHFR
- α_6: mean translocation time for reduced mDHFR minus time delay between mixing components and starting the luminescence measurement
- α_7: RMS deviation translocation time for reduced mDHFR
- α_8: furimazine substrate depletion rate
- α_9: scaling factor for instrument sensitivity
- α_{10}: relative change in luminescence reading after dilution (at T_G)
- α_{11}: relative change in luminescence reading after dilution (at T_R)

At $t = T_0$, the experiment is set up by mixing proteoliposomes with substrate and ATP. At $t = T_G$, GST-dark is added to the reaction. At $t = T_R$, DTT is added to the reaction. While $t < T_R$ (i.e. before DTT addition), the oxidized substrate protein engages with the translocon and is translocated until the disulfide loop. We consider the disulfide loop reduction after DTT addition to be essentially instantaneous. Thus, translocation resumes after reduction. The mean total time for substrate import into the interior of the proteoliposomes is the sum of mean time required for translocation (α_4) and the mean time required for unfolding ($1/\alpha_1$). The variation in translocation time between molecules is modeled as a Gaussian distribution with standard deviation of α_5 around the mean α_4. Unfolding is assumed to be a first-order process with rate α_1. When the substrate reaches the interior of the

proteoliposome, its C-terminal p86 tag restores NanoLuc activity by binding to the encapsulated 11S. Active NanoLuc uses furimazine substrate to generate light. As a consequence, the intra-liposome furimazine concentration decreases, which is captured by parameter α_8 as described below.

Not all substrate protein molecules are oxidized at $t = T_0$. As a consequence, full translocation can already occur at $t < T_R$. To account for the signal generated by bypassing disulfide loop stalling, we consider that a fraction ($1 - \alpha_3$) of the substrate protein is not oxidized. These molecules translocate into the interior of the vesicles (translocation time described by α_6 and α_7, unfolding described by α_1), starting at $t = T_0$, and contribute to the total amount of substrate protein inside proteoliposomes and to the overall luminescence signal.

When translocation is halted (either at the disulfide loop or at the folded mDHFR), irreversible "incapacitation" leads to irreversible loss of translocation activity. The molecular basis for incapacitation is not known. Possible processes include translocon inactivation, and substrate protein aggregation or sequestration. Incapacitation occurs with rate α_2 when translocation is stalled, resulting in lower overall signal. Because incapacitation occurs at a constant rate and competes with unfolding, the overall efficiency of translocation is scaled by a factor of $\alpha_1 / (\alpha_1 + \alpha_2)$.

The total time between DTT addition and appearance of the p86 tag inside the liposomes consists of three parts: the time τ_1 required for translocation to folded mDHFR, the time required for mDHFR unfolding, and the time τ_2 required for completing translocation, as shown in Supplementary Figure 2. Each of the three times varies from one molecule to another; the variation is described by probability distributions. The probability distribution for the total time is the convolution of the three probability distributions describing the individual components. Since the result of convolution is independent of the order of the parts, we can change the model by assuming that the unfolding of mDHFR is the first of the three parts, and it is followed by the translocation time $\tau_1+\tau_2$. This approach simplifies the model equations, but it has no effect on the result.

To calculate luminescence intensity, we define the functions $X_1(t)$, $Y_1(t)$, $Z_1(t)$, $X_2(t)$, $Y_2(t)$, and $Z_2(t)$, where the subscripts 1 and 2 refer to substrate proteins that were oxidized and reduced, respectively, at $t = T_0$. Functions $X_1(t)$ and $X_2(t)$ describe the molar fraction of pOA-DHFR molecules that are waiting for mDHFR to unfold and are still translocation competent at time t. Functions $Y_1(t)$ and $Y_2(t)$ describe the molar fraction of pOA-DHFR that have resumed translocation after mDHFR unfolding. Functions $Z_1(t)$ and $Z_2(t)$ describe the molar fraction of pOA-DHFR that have completed translocation.

Differential equations for $X_1(t)$ and $Y_1(t)$ and their solutions are

$$\begin{aligned} \frac{dX_1(t)}{dt} &= 0 && -\infty < t < T_0 \\ \frac{dX_1(t)}{dt} &= -\alpha_2 X_1(t) && T_0 \le t < T_R \\ \frac{dX_1(t)}{dt} &= -\alpha_1 X_1(t) - \alpha_2 X_1(t) && T_R \le t < +\infty \end{aligned} \tag{2}$$

$$
\begin{aligned}
\frac{dY_1(t)}{dt} &= 0 && -\infty<t<T_R \\
\frac{dY_1(t)}{dt} &= +\alpha_1 X_1(t) && T_R\le t<+\infty
\end{aligned} \tag{3}
$$

$$
\begin{aligned}
X_1(t) &= \alpha_3 && -\infty<t<T_0 \\
X_1(t) &= \alpha_3 \exp[-\alpha_2(t-T_0)] && T_0\le t<T_R \\
X_1(t) &= \alpha_3 \exp[-\alpha_2(T_R-T_0)-(\alpha_1+\alpha_2)(t-T_R)] && T_R\le t<+\infty
\end{aligned} \tag{4}
$$

$$
\begin{aligned}
&Y_1(t)=0 && -\infty<t<T_R \\
&Y_1(t)=\frac{\alpha_3\alpha_1}{\alpha_1+\alpha_2}\exp[-\alpha_2(T_R-T_0)]\{1-\exp[-(\alpha_1+\alpha_2)(t-T_R)]\} && T_R\le t<+\infty
\end{aligned} \tag{5}
$$

Differential equations for $X_2(t)$ and $Y_2(t)$ and their solutions are

$$
\begin{aligned}
\frac{dX_2(t)}{dt} &= 0 && -\infty<t<T_0 \\
\frac{dX_2(t)}{dt} &= -\alpha_1 X_2(t)-\alpha_2 X_2(t) && T_0\le t<+\infty
\end{aligned} \tag{6}
$$

$$
\begin{aligned}
\frac{dY_2(t)}{dt} &= 0 && -\infty<t<T_0 \\
\frac{dY_2(t)}{dt} &= +\alpha_1 X_2(t) && T_0\le t<+\infty
\end{aligned} \tag{7}
$$

$$
\begin{aligned}
X_2(t) &= (1-\alpha_3) && -\infty<t<T_0 \\
X_2(t) &= (1-\alpha_3)\exp[-(\alpha_1+\alpha_2)(t-T_0)] && T_0\le t<+\infty
\end{aligned} \tag{8}
$$

$$
\begin{aligned}
Y_2(t) &= 0 && -\infty<t<T_0 \\
Y_2(t) &= \frac{(1-\alpha_3)\alpha_1}{\alpha_1+\alpha_2}\{1-\exp[-(\alpha_1+\alpha_2)(t-T_0)]\} && T_0\le t<+\infty
\end{aligned} \tag{9}
$$

$Z_1(t)$ and $Z_2(t)$ are calculated as convolutions of $Y_1(t)$ and $Y_2(t)$ with corresponding translocation time distributions $P_n(\tau)$, where τ is the translocation time (τ equals τ_1 + τ_2, shown in Supplementary Figure 2):

$$Z_1(t) = \int_{-\infty}^{+\infty} Y_1(t-\tau)P_1(\tau)\,d\tau \tag{10}$$

$$Z_2(t) = \int_{-\infty}^{+\infty} Y_2(t-\tau)P_2(\tau)\,d\tau \tag{11}$$

Parameterization using Gaussian distributions for $P_n(\tau)$ gives:

$$P_1(\tau) = \frac{1}{\sqrt{2\pi\alpha_5^2}}\exp\left(-\frac{(\tau-\alpha_4)^2}{2\,\alpha_5^2}\right) \tag{12}$$

$$P_2(\tau) = \frac{1}{\sqrt{2\pi\alpha_7^2}}\exp\left(-\frac{(\tau-\alpha_6)^2}{2\,\alpha_7^2}\right) \tag{13}$$

As described above, α_4 is the mean translocation time for oxidized pOA-DHFR. α_6 is the mean translocation time for reduced pOA-DHFR minus the time delay between mixing components and starting the luminescence measurement. α_5 is the RMS deviation of

translocation time for oxidized pOA-DHFR. α_7 is the RMS deviation for the mean translocation time for reduced pOA-DHFR.

Luminescence intensity is directly proportional to $[Z_1(t)+Z_2(t)]S(t)$ where $S(t)$ is the concentration of the furimazine substrate at time t divided by the concentration at T_0. The depletion of substrate due to enzymatic activity can be described by:

$$\frac{d\, S_{\mathrm{DEPL}}(t)}{d\, t} = -\alpha_8 A(t) \tag{14}$$

S_{DEPL} describes the time dependence of relative furimazine concentration (relative to that at $t=T_0$), without taking into account dilutions at $t = T_{\mathrm{G}}$ and $t = T_{\mathrm{R}}$. $A(t)$ is the experimentally measured total luminescence intensity at time t. Parameter α_8 combines several unknown technical constants: n_{F}, the total number of furimazine molecules at $t=T_0$; η, the quantum yield of furimazine; Q, the quantum efficiency of the detection system (including the light collection system and the photomultipleir); φ, the fraction of time the photomultiplier counts photons from one well. A complete depletion of all furimazine molecules would produce the integral intensity $\int A(t)dt=\varphi\cdot Q\cdot\eta\cdot n_{\mathrm{F}}$, which corresponds to the decrease from 1 to 0 in S_{DEPL}, therefore $\alpha_8=1/(\varphi\cdot Q\cdot\eta\cdot n_{\mathrm{F}})$. Supplementary Equation (14) must be integrated numerically:

$$S_{\mathrm{DEPL}}(t_n) = 1 - \frac{\alpha_8}{2}\sum_{m=1}^{n}(A_{m-1}+A_m)(t_m-t_{m-1}) \tag{15}$$

The model function $I(t,\boldsymbol{\alpha})$, which is directly proportional to $[Z_1(t)+Z_2(t)]S(t)$:

$$I(t,\boldsymbol{\alpha}) = \alpha_9\,[Z_1(t)+Z_2(t)]\,S(t) \tag{16}$$

Here, α_9 is a scaling factor related to instrument sensitivity. α_9 is directly proportional to $\varphi\cdot Q\cdot\eta\cdot n_{\mathrm{F}}$ and also directly proportional to the concentration of pOA-DHFR at $t=T_0$ (the latter is because the functions $X_1(t)$, $Y_1(t)$, $Z_1(t)$, $X_2(t)$, $Y_2(t)$, and $Z_2(t)$ represent relative molar concentrations; to get the absolute concentrations these need to be multiplied by the initial concentration of pOA-DHFR). The values of the parameters α_8 and α_9 were expected to be the same for all wells that were read simultaneously, therefore during the analysis α_8 and α_9 were linked between the data sets.

Dilution and change in detection sensitivity between measurements (which could result from small variations in the exact positioning of the plate in the plate reader) are taken into account by α_{10} and α_{11}. These parameters report the ratio of the luminescence readings after and before the first and second dilution step (at $t = T_{\mathrm{G}}$ and $t = T_{\mathrm{R}}$, respectively).

Furimazine concentration at each of these time intervals is thus:

$$\begin{aligned} S(t) &= S_{\mathrm{DEPL}}(t) & -\infty<t<T_G \\ S(t) &= \alpha_{10} S_{\mathrm{DEPL}}(t) & T_G\le t<T_R \\ S(t) &= \alpha_{11} S_{\mathrm{DEPL}}(t) & T_R\le t<+\infty \end{aligned} \tag{17}$$

By fitting the model described above to the experimental data, we determined all values in the parameter vector $\boldsymbol{\alpha}$. Only α_8 and α_9 were treated as global parameters that were linked between data sets collected simultaneously. All other parameters were free to change during the fitting.

Supplementary Figure 2 provides a visual summary of the translocation model developed here. The parameter names in Supplementary Figure 2 relate to the parameter names in the description provided here as follows:

- $k_{\text{unfold}} = \alpha_1$
- $k_{\text{incap}} = \alpha_2$
- $(\tau_1 + \tau_2) = \alpha_4$

2. Numerical Methods of Fitting Luminescence Data

A FORTRAN program has been written to fit the kinetic model described above to the experimental data. The program is based on SUBROUTINE TOPFIT, which implements a general χ^2 minimum algorithm. In mathematical statistics χ^2 is defined as a random variable, however, in practice it is commonly used in connection with the chi-square goodness-of-fit test.[1] In the latter case the χ^2 value is calculated as the sum of squared differences between data and model, divided by corresponding variances:[1]

$$\chi^2 = \sum_{n=1}^{N_D} \frac{[D_n - M_n(\boldsymbol{\alpha})]^2}{V_n} \tag{18}$$

Here, N_D is the total number of data points; D_n is the experimental data value for point n; $M_n(\boldsymbol{\alpha})$ is a model prediction for point n, in our case $M_n(\boldsymbol{\alpha}) = I(t_n,\boldsymbol{\alpha})$, where $I(t,\boldsymbol{\alpha})$ is defined in Supplementary Equation (16) and t_n is the time for point n; V_n is the variance estimate for point n.

Variance estimates. Variance estimates V_n are calculated by the main program using the fact that photon counts obey Poisson statistics. The data D_n are obtained by by subtracting the background photon counts B_n from the total luminescence photon counts A_n,

$$D_n = A_n - B_n \tag{19}$$

The total luminescence photon counts A_n and background photon counts B_n are recorded quasi-simultaneously by a microplate reader; the well from which the background signal is derived contains all the same reagents except ATP. Since ATP is required for translocation, all the luminescence signal from the well without ATP results from background luminescence that needs to be subtracted. Since both A_n and B_n obey Poisson statistics, their variances equal their ensemble means, and since A_n and B_n are statistically independent, the variances add up:

$$V_n = \langle A_n \rangle + \langle B_n \rangle \tag{20}$$

The ensemble means, $\langle A_n \rangle$ and $\langle B_n \rangle$, are unknown, however, for large photon counts the difference between $\langle A_n \rangle + \langle B_n \rangle$ and $A_n + B_n$ becomes insignificant. Since in all our data sets $A_n > 10^5$ and $B_n > 10^5$ for every n, we used $A_n + B_n$ instead of $\langle A_n \rangle + \langle B_n \rangle$ for variance estimates.

Exclusion of outlier data points. At this stage the program excludes from the analysis a small number of data points that appear as spikes in the signal (see Supplementary Figure 3, open circles). Typically spikes in the signal from a photomultiplier (PMT) used in the

photon-counting mode could result (1) from bad vacuum inside the PMT tube, which is a result of prior abuse, (2) from a bad contact inside the PMT housing or in one of the connectors between the PMT anode and the amplifier, or (3) from cosmic rays. To eliminate the bad data points, the model is fit to one data set at a time (not global fitting) so that a good fit to the data is obtained. The parameter values that produced the good fit may be not unique, the only thing that matters is that the fit must be good upon visual inspection. Then weighted residuals are calculated using the formula $\mathrm{WR}_n=[D_n-M_n(\boldsymbol{\alpha})]/\sqrt{V_n}$. The threshold for data point exclusion is set at the square root of ten times the mean squared weighted residual, i. e., $\mathrm{WR}_{\mathrm{TH}}=\sqrt{10\langle \mathrm{WR}_n^2\rangle}$. The data points with $\mathrm{WR}_n>\mathrm{WR}_{\mathrm{TH}}$ are then excluded from the analysis. This method has a false positive rate of about 0.00157, i. e., on average about one out of 639 good data points gets rejected when no spikes are present. When the spikes are present, the number of rejected good data points is less then one out of 639 because the spikes increase the rejection threshold. The false negative rate depends on the origin of the spikes in the data, which is unknown, but visual inspection of the fits reveals that all spikes have been removed.

Calculation of the model function, its derivatives, and the Hessian matrix. A lower-level SUBROUTINE MODEL, written specifically for this data analysis, calculates the model function $M_n(\boldsymbol{\alpha})$ and its derivatives with respect to parameters α_i. Derivatives are calculated using analytical method rather than numerical finite differences method. SUBROUTINE MODEL is called multiple times from the SUBROUTINE TOPFIT, which calculates not only the χ^2, but also the the vector **V** and the Hessian matrix **H**, with the elements

$$V_i = \sum_{n=1}^{N_\mathrm{D}} \frac{1}{V_n} [D_n - M_n(\boldsymbol{\alpha})] \frac{\partial M_n(\boldsymbol{\alpha})}{\partial \alpha_i} \tag{21}$$

$$H_{ij} = \sum_{n=1}^{N_\mathrm{D}} \frac{1}{V_n} \left(\frac{\partial M_n(\boldsymbol{\alpha})}{\partial \alpha_i}\right) \left(\frac{\partial M_n(\boldsymbol{\alpha})}{\partial \alpha_j}\right) \tag{22}$$

The Hessian matrix **H** is then modified in accordance with the Marquardt algorithm,[2]

$$Q_{ij} = \begin{Bmatrix} (1+\lambda) H_{ij} & \text{if} & i=j \\ H_{ij} & \text{if} & i\neq j \end{Bmatrix} \tag{23}$$

where λ is the damping parameter, a small positive number (the diagonal elements of the matrix **H** get multiplied by a factor $1+\lambda$, while the off-diagonal elements are unchanged). The Marquardt-modified Hessian matrix **Q** is inverted, the inverse matrix is denoted $\mathbf{Q}^{-1}$. The correction vector $\boldsymbol{\delta}$ is then calculated by matrix multiplication

$$\boldsymbol{\delta} = \mathbf{Q}^{-1}\mathbf{V} \tag{24}$$

Iterative process. Iterations start from the vector $\boldsymbol{\alpha}^{(0)}$ of initial guesses and the initial damping parameter $\lambda^{(0)}$ that are passed to SUBROUTINE TOPFIT by the main program. We used $\lambda^{(0)}=10^{-8}$. In the first iteration ($m=0$) or a subsequent iteration number $m+1$ the correction vector $\boldsymbol{\delta}^{(m)}$ is computed from the parameter vector $\boldsymbol{\alpha}^{(m)}$ and the damping parameter $\lambda^{(m)}$ as per Supplementary Equations (21-24) and then used to compute the updated parameter vector $\boldsymbol{\alpha}^{(m+1)}$:

$$\boldsymbol{\alpha}^{(m+1)} = \boldsymbol{\alpha}^{(m)} + \boldsymbol{\delta}^{(m)} \tag{25}$$

Domain of tolerable values in parameter space. Once the new vector $\boldsymbol{\alpha}^{(m+1)}$ is obtained, SUBROUTINE TOPFIT calls LOGICAL FUNCTION DOMAIN, which is a match to SUBROUTINE MODEL. The FUNCTION DOMAIN returns the value TRUE if all the parameter values are within the tolerable domain and FALSE if $\boldsymbol{\alpha}$ is outside the tolerable domain. FUNCTION DOMAIN must return the FALSE value (i) if the execution of SUBROUTINE MODEL will result in an invalid operation, such as division by zero, overflow, logarithm of a negative number, etc., (ii) if the values of model parameters make no physical sense (like a negative reaction rate or a negative width of a distribution function), and (iii) (this is desirable rather than required) if the parameter values will result in a singular Hessian matrix. For the specific model considered here the following restrictions are imposed on parameter values: $\alpha_1>0$, $\alpha_2\geq0$, $0\leq\alpha_3\leq1$, $\alpha_5>0$, $\alpha_7>0$, $\alpha_8\geq0$, $\alpha_9\geq0$, $\alpha_{10}\geq0$, $\alpha_{11}\geq0$, no restrictions on α_4 and α_6.

Control of the damping parameter λ. The value of $\chi^2[\boldsymbol{\alpha}^{(m+1)}]$ is calculated and compared to $\chi^2[\boldsymbol{\alpha}^{(m)}]$ only if FUNCTION DOMAIN returns the TRUE value. In the following three cases: (A) if FUNCTION DOMAIN returned the FALSE value, or (B) if the matrix $\mathbf{Q}$ is not positive-definite, or (C) if $\chi^2[\boldsymbol{\alpha}^{(m+1)}] > \chi^2[\boldsymbol{\alpha}^{(m)}]$, the results of iteration $m+1$ are canceled, the value of $\lambda^{(m)}$ is multiplied by ν, where $\nu>1$ (specific value is discussed below), and the operations in Supplementary Equations (23-25) are repeated until (A) FUNCTION DOMAIN returns the TRUE value, (B) matrix $\mathbf{Q}$ is positive-definite, and (C) the condition $\chi^2[\boldsymbol{\alpha}^{(m+1)}] \leq \chi^2[\boldsymbol{\alpha}^{(m)}]$ is satisfied. If $\chi^2[\boldsymbol{\alpha}^{(m+1)}] \leq \chi^2[\boldsymbol{\alpha}^{(m)}]$, then the results of iteration $m+1$ are accepted and the next iteration begins; if the value of $\lambda^{(m)}$ did not have to be increased during iteration $m+1$, then $\lambda^{(m+1)}=\lambda^{(m)}/\nu$. If the value of $\lambda^{(m)}$ had to be increased during iteration $m+1$, then $\lambda^{(m+1)}=\lambda^{(m)}$. While Marquardt[2] originally proposed to use the value of 10 for ν, in miscellaneous previous applications of SUBROUTINE TOPFIT we found that the fastest convergence of Marquardt algorithm (the minimum number of iterations required to get to the minimum) is achieved with ν between 2 and 8, depending on the specific model and initial guesses, thus here we use $\nu=4$. The value of ν has absolutely no effect on the final values of model parameters and little effect on the ability of the algorithm to find the minimum.

Stopping criterion and false (apparent) χ^2 minima. The iterative process stops after iteration $m+1$ if all three of the following conditions are met at the same time:

(a) $\lambda^{(m)} \leq \lambda_T$, where λ_T is the threshold value of λ,

(b) $\boldsymbol{\delta}^{(m)}\cdot\mathbf{V}^{(m)} \leq \varepsilon\, \chi^2[\boldsymbol{\alpha}^{(m+1)}]$, where centered dot denotes the scalar product of two vectors,

(c) $\chi^2[\boldsymbol{\alpha}^{(m)}] - \chi^2[\boldsymbol{\alpha}^{(m+1)}] \leq \varepsilon\, \chi^2[\boldsymbol{\alpha}^{(m+1)}]$.

The threshold value of the of the damping parameter, λ_T, and the χ^2 accuracy parameter ε are passed to SUBROUTINE TOPFIT by the main program; both parameters must be positive. We used $\lambda_T=10^{-8}$ and $\varepsilon=10^{-8}$. The best value of λ_T depends on the degree of correlation between model parameters. When there is high correlation between model parameters, a very low value of λ_T is required to avoid false minima that result from the iterative process stopping before the true minimum is reached. If λ_T is sufficiently low for the given model, then, due to condition (a), the value of $\lambda^{(m)}$ is sufficiently low during the last iteration, in which case Marquardt algorithm becomes equivalent to Gauss-Newton algorithm, and the scalar product $\boldsymbol{\delta}^{(m)}\cdot\mathbf{V}^{(m)}$ gives an accurate prediction of the difference between the current χ^2 value and the minimum χ^2 value. Thus, a combination of conditions (a) and (b) assures that the current χ^2 value is no greater than $(1+\varepsilon)$ times the minimum χ^2 value. Condition (c)

is the least reliable of the three stopping criteria, yet, some least-squares programs rely on condition (c) alone for the stopping criterion, which is what results in false (apparent) minima that are then confused with true local minima.

Possible failures of the χ^2 minimization algorithm and importance of analytical derivatives. The accuracy of the final values of model parameters $\boldsymbol{\alpha}$ depends on the values of λ_T and ε: the lower these values of λ_T and ε, the more accurate the results. However, if the values of λ_T or ε are too low, then there is another problem: the algorithm never stops. The likelihood of this outcome depends on the accuracy of the derivatives calculated by SUBROUTINE MODEL. We employ analytical method for derivative calculation and REAL(8) arithmetic, which makes it possible to safely use $\lambda_T=10^{-8}$ and $\varepsilon=10^{-8}$. Most least-squares programs use numerical finite differences for derivative calculation, which is significantly less accurate and results in either failure to stop (with small ε) or stopping too soon (with large ε). The use of the analytical method for derivative calculation and the appropriate choice of λ_T and ε eliminates these problems.

Successful applications of SUBROUTINE TOPFIT for global analyses with challenging model functions have been reported.[3,4] No false minima or local mimima have been found in those applications. This does not mean that SUBROUTINE TOPFIT will find the χ^2 minimum with any initial guesses $\boldsymbol{\alpha}^{(0)}$. If the initial guesses are too far off, then the program outputs an error message, for example: "Initial guesses outside the domain of tolerated values in parameter space" or "Chi square gradient leads outside the domain of tolerated values in parameter space". This is considered a failure rather than a local minimum.

Models with multiple true local minima. There are known examples of models with either more than one discrete local χ^2 minimum in parameter space or a continuum of local χ^2 minima, which constitutes a subspace in multidimensional parameter space. An example of the first kind is the model function $f(t)=\alpha_1\exp(-t/\tau_1)+\alpha_2\exp(-t/\tau_2)$ that is often used to fit kinetic processes. Suppose that one true χ^2 minimum has been found. Swapping the values of α_1 with α_2 and τ_1 with τ_2 results in another true χ^2 minimum with the same χ^2 value. A linear combination of n exponentials results in $n!$ equivalent true χ^2 minima, which was practically observed with the program tcphoton[5] that is also based on SUBROUTINE TOPFIT. Which of the $n!$ minima tcphoton will find depends on the initial guesses. An example of the second kind is the model function $f(x)=\alpha_1 x+\alpha_2 x+\alpha_3$. Suppose that one true χ^2 minimum has been found. Any other set of parameters that has the same sum $\alpha_1+\alpha_2$ and the same value of α_3 results in the same χ^2, which means that there is a one-dimensional subspace (line) of local χ^2 minima in the three-dimensional parameter space.

The kinetic model described in section 1 of this Supplementary Information represents a clear example of the second kind. If the fitting is performed with one data set at a time, for example, only with the apo data or only with the MTX data or only with the MTX+NADPH data, then there is a one-dimensional subspace of equivalent χ^2 minima in the eleven-dimensional parameter space. More specifically, one can take any value of parameter α_2 (incapacitation rate) between 0 and the value slightly higher than the one in Supplementary Table 1, fix that value, and then by adjusting the values of the remaining 10 free model parameters the program will be able to find a local χ^2 minimum with exactly the same χ^2 value (the first eight significant digits are the same).

Eliminating the ambiguity by global analysis. As mentioned above, it is possible to fit all luminescence data with the value of parameter α_2 set to 0 and fixed. The fits are adequate, but the values of some model parameters make little sense. Specifically, the values of parameters α_9 (scaling factor for instrument sensitivity) differs by more than one order of magnitude between the apo, MTX, and MTX+NADPH samples, although all three samples were measured simultaneously in one instrument in the same microplate. Parameters α_9 and α_8 (furimazine substrate depletion rate) include several unknown factors, including initial furimazine concentration, the efficiency of the light collection system, and the sensitivity of the photomultiplier, but during one experiment all these unknown factors should be the same across all samples. This means that the data obtained with the three samples (apo, MTX, and MTX+NADPH) should be analyzed globally and the values of the global parameters α_8 and α_9 should be linked across all three sets, whereas the values of the remaining 11-2=9 local parameters should remain free. Now let us count the dimensions. 3 models were joined with 11 parameters each, this makes a 33-dimensional parameter space. In this space there were 3 dimensions of ambiguity (one per data set), i. e., there is a three-dimensional subspace of equivalent local χ^2 minima in the 33-dimensional parameter space. Linking two parameter across three data sets reduced the number of freedoms by 2×(3-1)=4, which exceeds the dimensionality of the subspace. Thus, it is likely that the resulting global model with 33-4=29 free parameters will have only one χ^2 minimum. Using a global version of the program described above, one true χ^2 minimum has been found. Now the question remains whether this is the only χ^2 minimum or there are other local χ^2 minima in the 29-dimensional parameter space.

Searching for alternative local minima. Iterative χ^2 minimization process can be compared to the flow of a river in a multidimensional terrain, in the sense that the χ^2, like water, can only go down, but it can not go up. This makes it possible to hypothetically divide the parameter space into drainage areas, with one drainage area per each local minimum. There are also drainage areas that do not drain into a local minimum, but rather take the parameter vector to the boundary of the domain in which the model function is defined; once the boundary is reached, the program will divide by zero or perform another illegal operation, which does not qualify as a local minimum. Thus, if the initial guess belongs to the drainage area that drains into a specific local minimum, then a χ^2 minimization will converge to that minimum and not any other. This suggests the following brute force approach to the search for alternative local minima. A random number generator is used to generate a wide range of physically possible values for each model parameter; then sets of randomly-generated parameters are used as initial guesses for global fitting. If a very large number of global fits with randomly-generated initial guesses is performed, then most of the local minima should be found.

In random generation of initial guesses different approaches were used for different parameters. The probability density of the initial guesses for parameters α_1, α_2, α_8, and α_9 was evenly distributed on the logarithmic scale over two orders of magnitude, from one tenth of the value of that parameter corresponding to the only known χ^2 minimum to ten times the value of that parameter corresponding to the only known χ^2 minimum. The probability density of the initial guesses for parameter α_3 was evenly distributed between 0.5 and 1.0 on a linear scale (theoretically this parameter can have values between 0 and 1,

but under our experimental conditions there is very little chance that α_3 could be less than 0.5). The probability density of the initial guesses for parameters α_4, α_5, α_6, and α_7 was evenly distributed between 50 s and 450 s on a linear scale. The probability density of the initial guesses for parameters α_{10} and α_{11} was evenly distributed between 0.8 and 1.0 on a linear scale.

Luminescence experiments were repeated four times, therefore there were four independent data sets for the global analyses (with each data set including one subset in the apo condition, one subset in the MTX-bound condition, and one in the MTX+NADPH-bound condition). In the global analysis of the data from each luminescence experiment we used 4096 randomly-generated sets of initial guesses, which makes the total of 16384 global analyses. We limited the number of initial guesses to this value because the global analysis requires significant computing time (approximately 193 hours for 16384 global analyses on an Intel quad-core 2.4GHz processor). Out of the 16384 sets of initial guesses 12268 (or about 74.9%) resulted in failures of the χ^2 minimization algorithm, which was reported by an error message (this is discussed on page 16). 4116 sets of initial guesses (or about 25.1%) converged to a true χ^2 minimum. For each of the four luminescence experiments there was only one χ^2 minimum; no other local minima were found. To establish whether the minima found in different global analyses were the same we compared the χ^2 values and the values of the parameters α_i . The χ^2 value output by the program contained 8 significant digits; all 8 digits were always the same, therefore the difference in the χ^2 value was less than 10^{-7} of the χ^2 value. Parameter value output contained 7 significant digits; for some parametes (α_6, α_9) the differences between the values were up to 4.11×10^{-4} of the value of that parameter, however, for most parameters it was much less than that. Therefore, there is no evidence of alternative local minima in our specific case.

<u>Variances estimates for the model parameters obtained from a single experiment.</u> After the χ^2 minimum is found, SUBROUTINE TOPFIT inverts the Hessian matrix **H** that was calculated in accordance with Supplementary Equation (22) during the last iteration. The inverted matrix **H** will be denoted **C**. Note, that **H** is not the same as **Q** and **C** is not the same as $\mathbf{Q}^{-1}$., therefore $\mathbf{Q}^{-1}$ (which has been calculated already) cannot be used instead of **C**. As it was shown elsewhere,[1] matrix **C** plays the role of the variance-covariance matrix for the values of the model parameters,

$$\langle(\alpha_i-\langle\alpha_i\rangle)(\alpha_j-\langle\alpha_j\rangle)\rangle = C_{ij} \tag{26}$$

SUBROUTINE TOPFIT saves on disk matrix **C** in binary format. Variances for the model parameters equal the corresponding diagonal elements,

$$\mathrm{var}(\alpha_i) = C_{ii} \tag{27}$$

Averaging the results from multiple experiments. The problem of calculating the most accurate mean estimate from several values, each with its own variance, is solved elsewhere;[1] we use the solution described there to calculate the mean value of each model parameter over the four experiments:

$$\overline{\alpha_i} = \left(\sum_{k=1}^{N_E} \frac{1}{\mathrm{var}(\alpha_{ik})} \right)^{-1} \sum_{k=1}^{N_E} \frac{\alpha_{ik}}{\mathrm{var}(\alpha_{ik})} \tag{28}$$

$$\mathrm{var}(\overline{\alpha_i}) = \left(\sum_{k=1}^{N_E} \frac{1}{\mathrm{var}(\alpha_{ik})} \right)^{-1} \tag{29}$$

where N_E is the number of experiments, in our case N_E=4, α_{ik} is the value of the model parameter α_i obtained in the analysis of the data from experiment k, var(α_{ik}) is the variance for α_{ik} calculated in accordance with Supplementary Equation (28), $\overline{\alpha_i}$ is the mean value of the model parameter α_i, and $\mathrm{var}(\overline{\alpha_i})$ is the variance of the mean value. This calculation of the mean parameters and the variances for the mean values assumes that the N_E experiments are completely reproducible and the only source of errors is the random noise (in our case, the fluctuations in the Poisson-distributed photon counts). To determine whether the experiments were not completely reproducible one can use the chi-squared test.[1] Reduced χ^2 can be calculated separately for each parameter α_i ,

$$\mathrm{reduced}\,\chi^2(\alpha_i) = \frac{1}{N_E - 1} \sum_{k=1}^{N_E} \frac{(\alpha_{ik} - \overline{\alpha_i})^2}{\mathrm{var}(\alpha_{ik})} \tag{30}$$

where N_E-1 is the number of degrees of freedom, defined as the difference between the number of α_{ik} values for the given i minus the number of parameters estimated from these values, in our case - one, $\overline{\alpha_i}$. The value of the reduced χ^2 is expected to be within certain limits, which depend on the number of degrees of freedom. For example, for N_E-1=3 degrees of freedom, the reduced χ^2 is expected to be less than 2.605 with 95% probability and less than 5.422 with 99.9% probability. The values of the reduced χ^2 calculated from our data are between 7.682 and 8637.0 for different α_i. This shows that some variations in experimental conditions (rather than the noise in the photon counts) were the main source of the errors in the parameter values. The variances calculated as per Supplementary Equation (29) have to be corrected by multiplying each variance by the corresponding reduced χ^2 obtained from Supplementary Equation (30), and then the standard deviations for the mean values $\overline{\alpha_i}$ can be obtained by taking square roots of the corrected variances,

$$\sigma(\overline{\alpha_i}) = \sqrt{\mathrm{var}(\overline{\alpha_i}) \times \mathrm{reduced}\,\chi^2(\alpha_i)} \tag{31}$$

Final parameter values and standard deviations reported in Table 1 were obtained as per Supplementary Equations (28) and (31), respectively.

Effects of changes in individual parameters on the quality of global fit. The quality of fit can be either inspected visually or judged based on the χ^2 value. We use the second approach because it is objective. In this section it is assumed that the χ^2 minimum has been found, the minimum χ^2 value is denoted $\chi^2_{\min}$ and the corresponding parameter values are denoted $\alpha_i^{\min}$. In the vicinity of the χ^2 minimum the first derivatives $\partial\chi^2/\partial\alpha_i$ equal zero

(this is the condition of the minimum), therefore the Taylor expansion of the χ^2 starts from the second-power term,[5]

$$\chi^2(\boldsymbol{\alpha}) = \chi^2_{\text{min}} + \sum_{i=1}^{N_P}\sum_{j=1}^{N_P} H_{ij}(\alpha_i-\alpha_i^{\text{min}})(\alpha_j-\alpha_j^{\text{min}}) + \ldots \tag{32}$$

Here H_{ij} is an element of the Hessian matrix defined in Supplementary Equation (22) and "..." denotes the third- and higher-power terms in $\boldsymbol{\alpha}$-$\boldsymbol{\alpha}^{min}$ that are significant only far away from the χ^2 minimum and can be omitted near the minimum. The next question is: what is the threshold for the χ^2 increase relative to the minimum that is considered acceptable? The threshold value can be expressed in terms of the F-distribution,[1,5]

$$\chi^2_T = \chi^2_{\text{min}} + \frac{N_S \cdot \chi^2_{\text{min}}}{N_D - N_P}\, F(p; N_S, N_D - N_P) \tag{33}$$

Here N_D and N_P are the number of data points and the number of free model parameters that have been determined from these data, N_D-N_P is the number of degrees of freedom, and N_S is number of parameters being forced away from their optimum values in order to increase the χ^2; if we consider the effect on the quality of fit of one parameter at a time, then N_S=1. For confidence areas in parameter space, N_S=2. For confidence volumes, N_S=3, etc.. The function $F(p;m,n)$ is the cumulative function for the F-distribution. The first argument of this function, p, represents the probability that the χ^2 does not exceed the threshold value χ^2_T . When F-distribution is used for calculation of confidence intervals, parameter p is included in the name of that confidence interval, for example, for a 95% confidence interval, p=0.95. Mathematically the function $F(p;m,n)$ is calculated by first solving Supplementary Equation (34) with respect to ξ and then substituting the value of ξ into Supplementary Equation (35):

$$\frac{B(\xi; m/2, n/2)}{B(m/2, n/2)} = p \tag{34}$$

$$F(p; m, n) = \frac{n\xi}{m(1-\xi)} \tag{35}$$

In Supplementary Equation (34) $B(\xi;a,b)$ denotes the incomplete beta-function and $B(a,b)$ denotes the complete beta-function:

$$B(\xi; a, b) = \int_0^{\xi} t^{a-1}(1-t)^{b-1}\,dt \tag{36}$$

$$B(a,b) = B(1; a, b) = \frac{\Gamma(a)\,\Gamma(b)}{\Gamma(a+b)} \tag{37}$$

where $\Gamma(x)$ is the gamma-function. Using Supplementary Equations (33-37) one can obtain the threshold value χ^2_T for any combination of p and N_D-N_P (N_S=1 in our case). This makes it possible to find the interval of possible values for every parameter α_i. Based on Supplementary Equation (32), it is tempting to do this using the following formula,

$$|\alpha_i - \alpha_i^{\text{min}}| \le \sqrt{\frac{\chi^2_T - \chi^2_{min}}{H_{ii}}} \tag{38}$$

But this is a flawed approach: Supplementary Equation (38) can be used only when there is no correlation between model parameters, i. e. when the Hessian matrix is diagonal. Correlation between model parameters results in the following problem: when one parameter α_i is deviated from its equilibrium value $\alpha_i^{\min}$, the remaining parameters adjust to minimize the χ^2, and the resulting increase in the χ^2 value is considerably less then it would be if only one parameter was allowed to change. The correlation between parameters can be taken into account by using Supplementary Equation (39) instead of Supplementary Equation (38).[5]

$$\left|\alpha_i - \alpha_i^{\min}\right| \le \sqrt{\left(\chi_T^2 - \chi_{min}^2\right) C_{ii}} \qquad (39)$$

Here C_{ii} is a diagonal element of the inverted Hessian matrix **C**, defined on page 18. Matrix **C** is also known as the variance-covariance matrix for parameter estimates.[1]

Supplementary Equation (39) has been used to obtain the intervals of parameter values that resulted in acceptable quality of global fits. The value of $p=0.999$ ensures that taking a parameter value outside the interval would result in a really bad fit, that could naturally occur only in one case out of a thousand. The 99.9% confidence intervals for the parameter that represents the unfolding rate of apo-mDHFR are: [3.104×10^{-4}, 3.459×10^{-4}], [4.317×10^{-4}, 4.695×10^{-4}], [4.165×10^{-4}, 4.471×10^{-4}], [4.400×10^{-4}, 4.732×10^{-4}] (where the four confidence intervals represent four different experiments). The 99.9% confidence intervals for the parameter that represents the unfolding rate of mDHFR in the presence of MTX and NADH are: [5.352×10^{-5}, 7.933×10^{-5}], [2.882×10^{-5}, 4.454×10^{-5}], [1.741×10^{-5}, 2.196×10^{-5}], [1.936×10^{-5}, 2.294×10^{-5}] (the four confidence intervals represent different experiments). The confidence intervals are narrow, which shows that a change in the value of parameter α_1 (unfolding rate of mDHFR) has a significant effect on the quality of fit, even when other parameters are free to adjust and compensate for the change in α_1. This is also true for most other parameters except for a few that are not of great importance to us (α_6, α_7, α_9). On the other hand, the four confidence intervals do not overlap, which shows once again that the main error resulted not from the photon-counting noise, but from reproducibility of experimental conditions. This problem was addressed above, see paragraph Averaging the results from multiple experiments on page 19.

3. Maximum Likelihood Estimation of Cossio-Hummer-Szabo Model Parameters

The force dependence of protein unfolding rates can be extracted from the distribution of unfolding forces obtained in force-ramp experiments. Here, we describe the approach that we used to analyze the unfolding force distributions of mDHFR in the presence of ligands. The approach described here was utilized to calculate the force dependence of the folded state lifetime and its standard deviation, shown in Figure 4. The calculated force-dependent unfolding rate was then related to the unfolding rate measured in translocation experiments (see Section 1 in Supplementary Information) to estimate the force generated by the translocon.

The rate of unfolding, k, for a folded protein in optical tweezers experiments is a function of the pulling force, F. How k varies with F depends on the properties of the molecule and the direction of applied force. The dependence of k on F and on the properties of the molecule was modeled using the formula developed by Cossio, Hummer and Szabo:[6]

$$k(F,\boldsymbol{\alpha}) \;=\; k_0\left(1-\frac{\nu F x^{\ddagger}}{\Delta G^{\ddagger}}\right)^{2-1/\nu}\exp\left\{\frac{\Delta G^{\ddagger}}{k_\mathrm{B} T}\left[1-\left(1-\frac{\nu F x^{\ddagger}}{\Delta G^{\ddagger}}\right)^{1/\nu}\right]\right\} \tag{40}$$

Here, $\boldsymbol{\alpha} = (k_0, x^{\ddagger}, \Delta G^{\ddagger}, \nu)$ represents the vector of model parameters. k_0 is the unfolding rate at zero force, $x^{\ddagger}$ is the distance from the native state energy minimum to the transition state, $\Delta G^{\ddagger}$ is the barrier height, and ν is a parameter that defines the shape of the free energy potential. Values of $\nu = 1/2$ or $\nu = 2/3$ are typically used. We obtained similar $k(F,\boldsymbol{\alpha})$ for both values of ν. The resulting values for the translocon-generated force are slightly lower for $\nu = 2/3$ ($\mathrm{F}_{\mathrm{mtx},\nu=1/2}$ = 10.6 [6.6, 13.2] pN, $\mathrm{F}_{\mathrm{mn},\nu=1/2}$ = 11.8 [10.6, 13.0] pN, $\mathrm{F}_{\mathrm{mtx},\nu=2/3}$ = 8.2 [5.0, 10.7] pN, $\mathrm{F}_{\mathrm{mn},\nu=2/3}$ = 5.5 [3.6, 7.1] pN). Thus, while a value of $\nu = 2/3$ yields a somewhat lower effective unfolding force (average: $\mathrm{F}_{\mathrm{avg}}$ = 6.9 pN) compared to the result obtained with $\nu = 1/2$ ($\mathrm{F}_{\mathrm{avg}}$ = 11.2 pN), it still indicates significant force generation by the translocon motor. As the fit was statistically better in the case of $\nu = 1/2$, we used this value of ν for our analyses.

The increase in pulling force F during a force ramp measurement is determined by the trap velocity and the total stiffness of the system. Instead of modeling the time variation of the pulling force, we determined it directly from the experimental data by fitting the following function to the data:

$$F(t) = \left[\left(a(t-t_0)^{p+r(t-t_0)}\right)^{-q} + \left(F_{\max}\right)^{-q}\right]^{-1/q} + c \tag{41}$$

This formula was found to adequately fit the force increase during ramp experiments for force-extension curves with and without DNA overstretching. Here, t_0 is the time when the ramp starts, a describes the slope of the ramp, p describes the curvature of the force ramp, r describes how the curvature changes, q describes the transition from the elastic response to overstretching of DNA, and $F_{\max}$ is the force at which overstretching is observed (~63 pN in our experiments). The force plateau during overstretching and its effect on the loading rate is therefore taken into account in our analysis. The parameter c describes the starting force of the ramp.

From Supplementary Equation (41), we obtained the first derivative $F'(t) = dF(t)/dt$ and an inverse function $t = F^{-1}(f)$, such that $F[F^{-1}(f)] = f$. This made it possible to express the time derivative of the force as a function of the force itself:

$$\dot{F}(f) \;=\; F'[F^{-1}(f)] \tag{42}$$

The distribution of rip forces was expressed in terms of the functions defined in Supplementary Equations (40) and (42):

$$\rho[F,\dot{F}(f),\boldsymbol{\alpha}] \;=\; \frac{k(F)}{\dot{F}(F)}\;\exp\left[-\int_0^F \frac{k(f)}{\dot{F}(f)}df\right] \tag{43}$$

To determine the unknown model parameters k_0, $x^{\ddagger}$, and $\Delta G^{\ddagger}$, the probability density in Supplementary Equation (43) must be fit to the experimental rip force data. In general, the

fitting can be done using either the method of nonlinear least squares (NLLS) or the method of maximum likelihood (MML). The use of NLLS requires binning the rip forces, where the choice of bin widths affect the result. Too wide bins result in the loss of force resolution, i.e., information loss. Too narrow bins result in small counts of rip events per bin, which hinders accurate variance estimation, because rip counts follow a Poisson distribution. When the total rip count in all bins is under 10000, there is no acceptable bin width that would result in at least 100 counts per bin and at least 100 bins per distribution width. Also, binning force rip data assumes that $\dot{F}(f)$ is exactly the same for all pulling curves, which is not the case for experimental data. The MML does not require binning or using an average $\dot{F}(f)$ function and is therefore free from the problems described above.

MML searches for the values of the unknown model parameters $\boldsymbol{\alpha}$ that maximize $L(\boldsymbol{\alpha})$, the likelihood of the parameter vector $\boldsymbol{\alpha}$ given the observed data:

$$L(\boldsymbol{\alpha}) = \prod_{n=1}^{N} \rho[F_n, \dot{F}_n(f), \boldsymbol{\alpha}] \tag{44}$$

Here N is the total number of observed rip events, F_n and $\dot{F}_n(f)$ are the rip force value and the force derivative function for the n-th rip event. The likelihood L is often an extremely small or extremely large number, outside of the range of 64-bit floating point numbers. It is therefore more convenient to deal with the natural logarithm of L,

$$\ln[L(\boldsymbol{\alpha})] = \sum_{n=1}^{N} \ln\{\rho[F_n, \dot{F}_n(f), \boldsymbol{\alpha}]\} \tag{45}$$

MML searches for the vector $\boldsymbol{\alpha}$ that maximizes $\ln[L(\boldsymbol{\alpha})]$. We used Newton's method to find the maximum. The method is briefly described below because one of the matrices involved in it also plays an important role in estimating the standard deviations for the model parameters and functions of these parameters. MML starts from an initial guesses vector $\boldsymbol{\alpha}^{(0)}$, and for this vector it calculates the elements of the vector $\mathbf{V}$ and the matrix $\mathbf{H}$,

$$V_i = \frac{\partial \ln[L(\boldsymbol{\alpha})]}{\partial \alpha_i} \tag{46}$$

$$H_{ij} = -\frac{\partial^2 \ln[L(\boldsymbol{\alpha})]}{\partial \alpha_i \partial \alpha_j} \tag{47}$$

The elements of the vector and the matrix are calculated by plain summation of the derivatives of $\ln(\rho_n)$ over all n; where ρ_n is obtained by substituting the rip force F_n and the corresponding $\dot{F}_n(f)$ in Supplementary Equation (43). The negative sign in Supplementary Equation (47) is necessary to make matrix $\mathbf{H}$ positive definite, at least in the vicinity of the maximum of $\ln[L(\boldsymbol{\alpha})]$. The correction to the vector of initial guesses is obtained by solving the matrix equation

$$\mathbf{H}\,\delta\boldsymbol{\alpha} = \mathbf{V} \tag{48}$$

The solution is obtained by first inverting the matrix $\mathbf{H}$. The inverted matrix is denoted $\mathbf{C}$. The correction vector is then calculated

$$\delta\boldsymbol{\alpha} = \mathbf{C}\mathbf{V} \tag{49}$$

and added to the initial guesses vector to yield the first iteration result:

$$\boldsymbol{\alpha}^{(1)} = \boldsymbol{\alpha}^{(0)} + \delta\boldsymbol{\alpha} \tag{50}$$

The result of the first iteration plays the role of the initial guesses for the second iteration, and so on. Iterations continue until the vector **V** becomes very close to the null-vector, which indicates that the maximum of $\ln[L(\boldsymbol{\alpha})]$ has been reached. Taylor expansion of $\ln[L(\boldsymbol{\alpha})]$ in the vicinity of its maximum is

$$\ln[L(\boldsymbol{\alpha})] = \ln(L^{(\max)}) - \frac{1}{2}\sum_{i=1}^{M}\sum_{j=1}^{M} H_{ij}\left(\alpha_i - \alpha_i^{(\max)}\right)\left(\alpha_j - \alpha_j^{(\max)}\right) + \ldots \tag{51}$$

where M is the number of unknown parameters and "..." denotes the terms of the third and higher powers in $\boldsymbol{\alpha}$-$\boldsymbol{\alpha}^{(\max)}$, which, according to the central limit theorem, lose significance as N increases. When the number of rips N is large, the higher-power terms can be omitted, and then exponential function can be taken of both sides of Supplementary Equation (51), which yields

$$L(\boldsymbol{\alpha}) = L^{(\max)} \exp\left[-\frac{1}{2}\sum_{i=1}^{M}\sum_{j=1}^{M} H_{ij}\left(\alpha_i - \alpha_i^{(\max)}\right)\left(\alpha_j - \alpha_j^{(\max)}\right)\right] \tag{52}$$

Note, that this is a general M-dimensional Gaussian distribution, from which it directly follows that the inverted matrix **H**, which above was denoted **C**, is the variance-covariance matrix for the estimates of the unknown model parameters. This means that the matrix **H** plays in MML exactly the same role that is played by the Hessian matrix in NLLS. The matrix **C** that was obtained as a byproduct from the last iteration makes it trivial to estimate the standard deviations of the model parameters and any functions of these parameters. The standard deviation for any model parameter equals the square root of the corresponding diagonal element of **C**,

$$\sigma\alpha_i = \sqrt{C_{ii}} \tag{53}$$

Now consider an arbitrary function Ψ (with continuous first derivatives) of the independent variable F and the model parameters $\boldsymbol{\alpha}$. The standard deviation for this function can be found by linearizing it in the vicinity of $\boldsymbol{\alpha}^{(\max)}$,

$$\sigma\Psi(F,\boldsymbol{\alpha}) = \sqrt{\sum_{i=1}^{M}\sum_{j=1}^{M} C_{ij}\left(\frac{\partial\Psi(F,\boldsymbol{\alpha})}{\partial\alpha_i}\right)\left(\frac{\partial\Psi(F,\boldsymbol{\alpha})}{\partial\alpha_j}\right)} \tag{54}$$

This approach was used to calculate the standard deviation for $\ln[\tau(F,\boldsymbol{\alpha})]$. Note, that $\ln[\tau(F,\boldsymbol{\alpha})]=-\ln[k(F,\boldsymbol{\alpha})]$, and $k(F,\boldsymbol{\alpha})$ is defined in Supplementary Equation (40).

Supplementary References

1 Hamilton, W. C. *Statistics in Physical Science: Estimation, Hypothesis Testing, and Least Squares.* (Ronald Press, New York, 1964).

2 Marquardt, D. W. An algorithm for least-squares estimation of nonlinear parameters. *J. Soc. Indust. Appl. Math.* **11**, 431-441 (1963).

3 Toptygin, D. Svobodova, J. Konopasek, I. & Brand, L. Fluorescence decay and depolarization in membranes. *J. Chem. Phys.* **96**, 7919-7930 (1992).

4 Toptygin, D, Chin, A. F. & Hilser, V. J. Effect of diffusion on resonance energy transfer rate distributions: implications for distance measurements. *J. Phys. Chem. B* **119**, 12603−12622 (2015).

5 Toptygin, D. Analysis of time-dependent red shifts in fluorescence emission from tryptophan residues in proteins. in *Fluorescence Spectroscopy and Microscopy: Methods and Protocols* (ed. Engelborghs, Y, Visser, AJWG) 215-256. *Methods in Molecular Biology* **1076** (2014).

6 Cossio, P, Hummer, G, & Szabo, A. Kinetic ductility and force-spike resistance of proteins from single-molecule force spectroscopy. *Biophys. J.* **111**, 832–840 (2016).
