# Supplementary material for: The SecA motor generates mechanical force during protein translocation
Source: Nat Commun. 2020 Jul 30;11:3802. doi: 10.1038/s41467-020-17561-2 (PMC7393111; doi:10.1038/s41467-020-17561-2)
Supplement: Supplementary file 2 — Peer Review File [file 41467_2020_17561_MOESM2_ESM.docx]

Peer Review Information

**Journal:** Nature Communications

**Manuscript title:** The SecA motor generates mechanical force during protein translocation

**Corresponding author name(s):** Dr Christian Kaiser

**Editorial notes:**

**none**

**Reviewer comments & decisions:**

**Reviewer comments, first version:**

Reviewer #1 (Remarks to the Author):

The research reported is excellent and the manuscript is
illuminating. Understanding the mechanics of translocation of
proteins across lipid bilayers is important but not studied sufficiently yet.
The authors explain how structured proteins can translocate through
the bilayers by first unfolding and then reconstituting.
The study is done by using a chimeric substrate: proOmpA-mDHFR.

I would only take an issue with the statement on p.7 in the context of
ref. 17. The correct and safe statement is that the stalling force for
ClpX is about 20 pN. Inferring that ClpX generates a force of
"up to 20 pN of mechanical force" is an overinterpretation
in my opinion. ClpX generats both axial force and a torque.
When a protein is pulled in it presses on ClpX which generates
a reaction force and, in the axial direction, one measures
the difference in the forces. This point is discussed in:
M. Wojciechowski et al. Biophys. J. 107:1661 (2014); Mol. BioSyst. 12:2700 (2016).


Reviewer #2 (Remarks to the Author):

The authors of this manuscript elegantly combine two experimental modalities (synchronized translocation luminescence experiments and single-molecule force spectroscopy) to estimate the mechanical force exerted by SecA to pull a protein through the translocon. Their method consisted essentially of two steps. First, they used a curve-fitting analysis of the translocation data to extract the unfolding rate of mDHFR preceding translocation. Then, they correlated this rate with force-dependent unfolding rates obtained from the force spectroscopy. They argued that the force exerted by SecA must be the force needed to cause mDHFR to unfold at the rate seen in the translocation study. Their result speaks to the mechanism of action of SecA, a vitally important protein across many biological systems about which there are fundamental open questions.

The elegance of the work, the significance of the results, and the clarity of the presentation combine to make this paper well suited for publication in Nature Communications. I believe it should be published as soon as the authors address one major concern related to the translocation curve-fitting analysis, as well several more-minor issues.

The main issue that the authors need to more fully explain is the procedure by which k_unfold was obtained from fitting the data of Fig. 1. The authors clearly explain in the SI the model they fit to these data. They should similarly provide 1) details of how the fitting was carried out, including how they could be sure they had reached global, rather than local, minima when fitting so many parameters at the same time and 2) a sensitivity analysis of how changes in the parameter values manifest in changes in the fitted curve. When fitting a many-parameter model like this, there is always the concern that the value of a particular parameter is free to change substantially with little effect on the fit curve; this would mean that the experiment is not very sensitive to that parameter.

Beyond this issue, there are a few minor points that should be addressed:

1. Why do the authors think the uncertainties in k_transloc are so small in Table 1? Are they underestimated? Or do the translocation rates actually disagree? (Also, there is a problem with the number of significant figures given in this table).
2. There is an argument made that apo mDHFR populates multiple slowly interconverting native states. The authors should sharpen this argument, which rests on interpreting a small number of points in the high-force tail of the rupture-force distribution and on particular parameter choices in making the plot of Fig. S5A. How many points are actually in this tail? And is there a way to make this argument general so that it does not depend on particular parameters in Fig. S5A? How well has this instrument generated skewed force ruptures that are well fit by the Dudko model. It should be noted that the Bell-evans model also predicts a skewed distribution.
3. The authors mention that a benefit of using the model of Dudko and co-workers is that it allows extrapolation to “obtain unfolding rates in force ranges that are not directly accessible in pulling experiments”. Is this extrapolation meaningfully altered by the choice to use nu = 1/2 in this model, compared with the alternative of nu = 2/3?
4. Does the use of biotin, which has a low rupture force compared to a covalent bond, bias the rupture-force distributions at high force? The authors may also want to comment on the overstretching transition preventing them from measuring rupture forces over 65 pN.
5. Why was 0.65 nm chosen as the persistence length for the worm-like chain model?
6. Why was the apo condition not used as a third data point in the analysis of Fig. 4?
7. In Table ST1, am I correct to understand that the standard deviations are based on averaging data across different molecules? If so, this should be clarified in the caption.
8. The authors should move a mention of references 43 and 45 into the introduction to appropriately recognize these works as exploring the mechanical stability of DHFR and its sensitivity to ligand binding (even if they came to opposite conclusions). Conceptually, they are looking at and modulating the mechanical stability of a protein that is unfolded by going through a pore. It doesn’t take away the novelty of the combination of biochemical and single molecule assays presented here for the key protein SecA. Indeed, reference 43 anticipated the elegant work demonstrated here when they stated “Therefore, if the rate-determining step in protein translocation were the force-induced unraveling of a protein, then our results would explain why the MTXDHFR complex is resistant to translocation”
9. There are multiple places where the text if figures is too small to read on a printed page (at least for an older persons eyes). For instance, I can’t read the subscripts on k in Figure 1D and also couldn’t distinguish the gray curve on top of the orange line. I had to blow it up on my monitor. The data is lovely, don’t make people work so hard to see it. I also couldn’t read the grey text in the legend of Figure 2D.
10. When transforming a rupture force distribution onto a k(F) vs F plot, the experimental data falls onto a portion of that plot (rather than the curve used in the present manuscript). What force range was that and did and how far did the authors need to extrapolate from their data to the regime where they are interpreting the data at 10 pN. To be clear, I am thinking of multitude of loading rates similarly transformed shown in a recent review (Figure 6D, Woodside and Block, Annu Rev Biophysics 2014). I am not asking the authors to take data rather clarify if there experimental data is needed to be extrapolated in the k(F) vs F graph.

**Author rebuttal, first version:**

We thank the reviewers for their detailed and constructive comments. Reviewer comments are in *Times New Roman font, italicized*. Our response to the individual points is inserted Arial font, with changes/insertions to the manuscript highlighted in red.

*Reviewer #1 (Remarks to the Author):*

*The research reported is excellent and the manuscript is illuminating. Understanding the mechanics of translocation of proteins across lipid bilayers is important but not studied sufficiently yet. The authors explain how structured proteins can translocate through the bilayers by first unfolding and then reconstituting. The study is done by using a chimeric substrate: proOmpA-mDHFR.*

We thank the reviewer for the succinct summary of our manuscript and the positive feedback.

*I would only take an issue with the statement on p.7 in the context of ref. 17. The correct and safe statement is that the stalling force for ClpX is about 20 pN. Inferring that ClpX generates a force of "up to 20 pN of mechanical force" is an overinterpretation*

*in my opinion. ClpX generats both axial force and a torque. When a protein is pulled in it presses on ClpX which generates a reaction force and, in the axial direction, one measures the difference in the forces. This point is discussed in: M. Wojciechowski et al. Biophys. J. 107:1661 (2014); Mol. BioSyst. 12:2700 (2016).*

We agree with the reviewer that the single-molecule experiments with ClpX yield the stalling force, and that the statement in our Discussion section was not accurate. As pointed out by the reviewer and explained in the Wojciechowski reference, pulling a folded protein (GFP in the case of the reference that we cited) against the ClpX rim generates a reaction force. The motor force is the sum of this reaction force and force applied by the tweezers. The stall force determined in the ClpX optical tweezers assay is therefore smaller than the motor force. We have included this aspect in the discussion and point the reader to one of the references (reference 54 in the revised manuscript):

“The stalling force of the dedicated protein unfoldase ClpX, which enables regulated protein degradation in the cell, was found to be approximately 20 pN in single-molecule optical tweezers experiments ^17^, although modeling indicates that the actual motor force may be considerably higher than the force that is externally applied in this assay ^54^.” (Discussion, p. 8)

*Reviewer #2 (Remarks to the Author):*

*The authors of this manuscript elegantly combine two experimental modalities (synchronized translocation luminescence experiments and single-molecule force spectroscopy) to estimate the*

1

Gupta *et al.* – Response

*mechanical force exerted by SecA to pull a protein through the translocon. Their method consisted essentially of two steps. First, they used a curve-fitting analysis of the translocation data to extract the unfolding rate of mDHFR preceding translocation. Then, they correlated this rate with force-dependent unfolding rates obtained from the force spectroscopy. They argued that the force exerted by SecA must be the force needed to cause mDHFR to unfold at the rate seen in the translocation study. Their result speaks to the mechanism of action of SecA, a vitally important protein across many biological systems about which there are fundamental open questions.*

*The elegance of the work, the significance of the results, and the clarity of the presentation combine to make this paper well suited for publication in Nature Communications. I believe it should be published as soon as the authors address one major concern related to the translocation curve-fitting analysis, as well several more-minor issues.*

We thank the reviewer for the positive comments.

*The main issue that the authors need to more fully explain is the procedure by which k_unfold was obtained from fitting the data of Fig. 1. The authors clearly explain in the SI the model they fit to these data. They should similarly provide 1) details of how the fitting was carried out, including how they could be sure they had reached global, rather than local, minima when fitting so many parameters at the same time and 2) a sensitivity analysis of how changes in the parameter values manifest in changes in the fitted curve. When fitting a many-parameter model like this, there is always the concern that the value of a particular parameter is free to change substantially with little effect on the fit curve; this would mean that the experiment is not very sensitive to that parameter.*

The reviewer points out an important concern for fitting complex models to experimental data. We have added a new section to the Supplementary Information (“2. Numerical Methods of Fitting Luminescence Data”; pages 12 to 20 in the revised document) that explains the fitting of the model in full detail. This section also describes extensive calculations that we have carried out in response to the reviewer’s concern. We believe that these explanations and calculations demonstrate that 1) the analysis yields a global (rather than a local) minimum regardless of the choice of initial parameter guesses, and that 2) the fit is sensitive to changes in the relevant parameters.

(1) The full details of the new calculations are described in the revised SI document. Briefly, we randomly generated 16384 sets of initial parameter guesses that covered a wide range of physically meaningful values for each individual parameter (e.g., values for the mDHFR unfolding rate, alpha_1, that ranged from 0.1 to 10 times the previously determined value). Each set of initial guesses was used to globally fit the three data sets (apo, MTX and M+N) simultaneously as described. (We refrained from even more extensive testing because the analysis required significant computational time). The algorithm failed to find a minimum in approximately 75% of attempts (likely because many of the starting points were separated from the global minimum by a boundary where the Hessian matrix is singular). In the remaining 25% of attempts, we obtained the same Chi square minimum, as defined by having a relative difference in Chi square

2

Gupta *et al.* – Response

values less than 10^-7^ and relative differences between parameter values less than 5 x 10^-4^. The observation that only one minimum is found for a wide range of initial parameter guesses indicates the absence of local minima.

(2) We also included a description of how sensitive the fit is to changes in individual parameters (“2. Numerical Methods of Fitting Luminescence Data”; pages 19 and 20 in the revised document). We show that changes in any of the parameters relevant for our study significantly lower the quality of the fit, even if the other parameters are free to adjust and compensate for the change. The fit is therefore sensitive to small changes in most parameters, giving us confidence that the results obtained in our analyses are meaningful.

*Beyond this issue, there are a few minor points that should be addressed:*

1. *Why do the authors think the uncertainties in k_transloc are so small in Table 1? Are they underestimated? Or do the translocation rates actually disagree? (Also, there is a problem with the number of significant figures given in this table).*

We are grateful that the reviewer points out this aspect. Our approach for calculating the uncertainties was indeed flawed in this case. The numbers reported in Table 1 of the original manuscript were calculated taking into account only the fluctuations in the Poisson-distributed photon counts. However, we realized that we also need to take into account variations between independent replicates of the luminescence measurements. This aspect is now described in detail in the revised SI document (“Averaging the results from multiple experiments.” in Section 2, p. 18). The means of the parameters remain unaffected by the revised calculations. The newly calculated uncertainties are reported in Table 1 and Supplementary Table ST1 of the revised manuscript. We believe that these values now more accurately reflect the uncertainty of our analyses.

2. *There is an argument made that apo mDHFR populates multiple slowly interconverting native states. The authors should sharpen this argument, which rests on interpreting a small number of points in the high-force tail of the rupture-force distribution and on particular parameter choices in making the plot of Fig. S5A. How many points are actually in this tail? And is there a way to make this argument general so that it does not depend on particular parameters in Fig. S5A? How well has this instrument generated skewed force ruptures that are well fit by the Dudko model. It should be noted that the Bell-evans model also predicts a skewed distribution.*

The reviewer raises a valid point. We interpreted our experimental results in the context of studies that have demonstrated the presence of multiple distinct native states for *E. coli* DHFR (reference 47 in the original manuscript, 50 in the revised version). Mouse DHFR has also been found to exist in two native conformers (Thillet *et al.* 1990, PMID 1974147). We have included this reference in Results section of the main text (number 49 in the revised manuscript). The relevant sentence now reads:

3

Gupta *et al.* – Response

“Given the distinct distributions observed for initial and subsequent events (Figure 2F), our results likely reflect the population of several native states, which has been observed for mDHFR ^49^ and for the orthologous *E. coli* enzyme ^50^.”

The simulations shown in Suppl. Fig. S5A are by no means a conclusive demonstration that the apo-mDHFR states have distinct mechanical stabilities. We included this figure as an illustration of the effect that such state switching-behavior would be expected to have on the force distributions from pulling experiments. We have revised the figure title to emphasize this point. It now reads “Illustration of the effects of state switching on unfolding force distributions”. We also added the following sentences to the figure caption:

“The parameters for simulating N1 and N2 unfolding were chosen to illustrate how state-switching manifests in unfolding force distributions. They do not reflect the true parameters of apo-mDHFR unfolding, which cannot be reliably determined from the experimental data described in this study.”

Regarding the question of the number of points in the high-force tail, we calculated at what force molecules in state N1 have unfolded with a probability of p = 0.99. This force is 48.9 pN for the parameters used. In the mixed distribution of N1 and N2, 7 out of 100 simulated unfolding forces occur at forces that exceed this force. While this number is relatively small, these events are easily identified because they fall into a region of the distribution where the integrated probability of unfolding (from F = 48.9 pN to infinity) from N1 is only 0.01. Since the true parameters for mDHFR unfolding are unknown, we cannot do the same calculation for the experimental data.

The instrument used in our study has indeed been previously used by us and others to generate skewed force distributions that are well described by the Dudko model (for examples, see Kaiser *et al.* 2011, PMID 22194581; Liu *et al.* 2019, PMID 31776255; Hao *et al.* 2019, PMID 31484930). The tail in the unfolding force distributions of apo-mDHFR is therefore not likely to be an artefact from the instrument used here.

Lastly, the reviewer correctly points out that the Bell-Evans model also predicts a skewed unfolding force distribution. We have included a reference to Evans and Ritchie (PMID 9083660; reference 47 in the revised manuscript) in the Results section of the main text:

“[...] the continuously increasing unfolding rate results in a characteristic skewed distribution of the unfolding force ^47,48^ [...]”

*3. The authors mention that a benefit of using the model of Dudko and co-workers is that it allows extrapolation to “obtain unfolding rates in force ranges that are not directly accessible in pulling experiments”. Is this extrapolation meaningfully altered by the choice to use nu = 1/2 in this model, compared with the alternative of nu = 2/3?*

4

Gupta *et a!.* – Response

A fit using nu = 1/2 yielded a statistically better fit to the experimental data. The sum of the logarithm of likelihoods (SLL) for the two nu values is SLL(nu = 1/2) = -774.1 and SLL(nu = 2/3) = -776.8. Comparing these two values yields exp(SLL(nu = 2/3) – SLL(nu = 1/2) = 0.067. A fit with nu = 1/2 therefore appears to describe the data better, which is why we chose to use this value for our analysis.

The results are similar for these two choices of nu and yield translocon forces in similar regions. While a parameter of nu = 2/3 yields slightly lower forces, it does not change the overall conclusion. However, we are now reporting these values in the Supplementary Information (Section 3, page 21), and we have inserted the following statement in the SI document:

“We obtained similar k (F ,α )for both values of ν. However, as the fit was statistically better in the case of ν = 1/2, we used this value of ν for our analyses. The resulting values for the translocon-generated force are slightly lower for nu = 2/3 (Fmtx, flu = 1/2 = 10.6 [6.6, 13.2] pN, Fmfl, flu = 1/2 = 11.8 [10.6, 13.0] pN, Fmtx, flu = 2/3 = 8.2 [5.0, 10.7] pN, Fmfl, flu = 2/3 = 5.5 [3.6, 7.1] pN) . Thus, while a value of nu = 2/3 yields a somewhat lower effective unfolding force (average: Favg = 6.9 pN) compared to the result obtained with nu = 1/2 (Favg = 11.2 pN), it still indicates significant force generation by the translocon motor.”

*4. Does the use of biotin, which has a low rupture force compared to a covalent bond, bias the rupture-force distributions at high force? The authors may also want to comment on the overstretching transition preventing them from measuring rupture forces over 65 pN.*

The reviewer raises another valid point. The biotin-streptavidin interaction indeed is likely the weakest link in our molecular assembly. Breaking of this linkage before mDHFR unfolding would skew the unfolding force distribution to lower values, which would lead to underestimating the mechanical stability of the protein (and,

consequently, the SecA motor strength). However, we consider this scenario unlikely, as explained in the following paragraphs.

The force-dependent dissociation of the biotin-streptavidin complex has been shown to strongly depend on the pulling geometry (see, e.g., Sedlak *et a!*. 2020, PMID 32232150, for an elegant recent study). We estimated the most likely rupture forces (FR) for our loading rate, using the Bell-Evans parameters from Sedlak et al. For simplicity, we assumed a constant loading rate of 15 pN/s, based on the trap speed of 150 nm/s and a trap stiffness of ~0.1 pN/nm (which should be the dominant contribution to overall stiffness in the relevant force range). For the three geometries that were experimentally tested by Sedlak et al., we obtain FR values of 18 pN, 0 pN and 241 pN. While the first two values are so low that we would not be able to actually measure mDHFR unfolding, the third value appears very high. This result might suggest either that our pulling geometry is similar to the “high stability” geometry, or that it is significantly different from the ones that were quantitatively characterized by Sedlak et al.

5

Gupta *et a!.* – Response

The most likely unbinding force for the pulling geometry employed in our experiments has not been determined, to our knowledge. However, the stability appears to be sufficient to reliably detect mDHFR unfolding, because we observed a clear unfolding transition in almost all of the “first pull” traces that were quantitatively analyzed in the manuscript. (Traces without a clear unfolding transition were occasionally observed in subsequent pulls, indicating the inefficient refolding of mDHFR described in the manuscript). We therefore think that the use of biotin-streptavidin in our experiments does not meaningfully bias the rupture force distributions.

Some of the events (4 out of 61 events) in the double-ligand condition obtained at the higher trap speed of 150 nm/s fall into force range of DNA overstretching (about 63 to 65 pN in our experiments). At this force, mDHFR unfolding is very rapid, and in none of the measurements did the folded state survive during full DNA overstretching. We therefore did not observe unfolding forces higher than the overstretching force.

The DNA overstretching transition does, however, affect the unfolding force distribution. When the force plateaus during DNA overstretching, the derivative of the force (Fdot of F) drops. This effect is taken into account in our analyses, because we used the experimentally measured loading rate for each individual force-extension curve (as described in Supplementary Information, Section 3). The results obtained in this way are therefore not affected by the DNA overstretching transition. We have inserted the following sentence (highlighted here in red) into the Supplementary Information (p. 21) to point out this aspect:

“[...] Fmax is the force at which overstretching is observed (~63 pN in our experiments). The force plateau during overstretching and its effect on the loading rate is therefore taken into account in our analysis.”

5. *Why was 0.65 nm chosen as the persistence length for the worm-like chain model?*

Persistence length values for unfolded polypeptides are generally in the range of 0.5 nm to 0.75 nm. A value of 0.65 nm is commonly used (see, for example, Shank *et a!.* 2010, PMID: 20495548; Yu *et a!.* 2012, PMID 22421432). We have used this value here because it appears to describe unfolded mDHFR well.

6. *Why was the apo condition not used as a third data point in the analysis of Fig. 4?*

As outlined in the response to point 2 (above), mDHFR appears to populate multiple native states with distinct mechanical stabilities. The unfolding forces are therefore a non-linear combination of two distributions that cannot be reliably resolved using the Dudko-based maximum likelihood approach that we employed for our analyses. We therefore cannot determine the kinetic parameters for unfolding of the apo protein. To better point out this aspect, we made the following changes/additions to the text:

*6*

Gupta *et al.* – Response

“Unfolding from multiple states yields unfolding forces that are a combination of at least two distributions that cannot be reliably resolved. The apparent structural heterogeneity of apo-mDHFR therefore hampers a quantitative analysis of its mechanical properties.” (Results, *“mDHFR is mechanically stable”*, p. 6)

“We developed a maximum-likelihood method for analyzing unfolding force distributions of the single (MTX) and double (M+N) ligand conditions (see Materials and Methods, and Supplementary Information). The presence of several native states in apo mDHFR, which cannot reliably be characterized in our experiments, precluded a similar analysis for this data set.” (Results, *“SecA actively promotes unfolding”*, p. 7)

7. *In Table ST1, am I correct to understand that the standard deviations are based on averaging data across different molecules? If so, this should be clarified in the caption.*

The standard deviations in Table ST1 were calculated from independent measurements of luciferase activity in our bulk translocation assay. As described in our response to point 1 (above), we have revised how the standard deviations were calculated and described the procedure in Supplementary Information. We made the following change to the table caption to point the reader to these calculations:

“See Supplementary Information, section "1. Kinetic Model for Fitting Luminescence Data" for a detailed description of the model and section “2. Numerical Methods of Fitting Luminescence Data” for calculation of means and standard deviations from independent replicates of the ensemble luminescence measurements.“

8. *The authors should move a mention of references 43 and 45 into the introduction to appropriately recognize these works as exploring the mechanical stability of DHFR and its sensitivity to ligand binding (even if they came to opposite conclusions). Conceptually, they are looking at and modulating the mechanical stability of a protein that is unfolded by going through a pore. It doesn’t take away the novelty of the combination of biochemical and single molecule assays presented here for the key protein SecA. Indeed, reference 43 anticipated the elegant work demonstrated here when they stated “Therefore, if the rate-determining step in protein translocation were the force-induced unraveling of a protein, then our results would explain why the MTXDHFR complex is resistant to translocation”*

We agree that it is useful to point to the atomic force microscopy studies in the introduction. We have therefore inserted the following paragraph into the introduction, which includes some content that was previously in the “Results” section (the reference numbers for the atomic force microscopy studies were 43 and 45 in the original version, and are 29 and 30 in the revised version):

“Dihydrofolate reductase (DHFR) has been widely used to study mitochondrial protein import ^22-25^ and Sec-dependent translocation ^7,26.^ The natural substrates of the enzyme,

*7*

Gupta *et a!.* – Response

dihydrofolate and nicotinamide adenine dinucleotide phosphate (NADPH), and the inhibitor methotrexate (MTX) have been shown to increase the thermodynamic stability of mammalian DHFR ^23,27,28.^ The protein thus functions as a translocation roadblock with tunable stability. Atomic force spectroscopy experiments exploring the mechanical effects of ligand binding came to opposite conclusions, suggesting stabilization of either the native state of the enzyme ^29^ or an unfolding intermediate ^30^. Quantitatively defining the mechanical resistance of DHFR in the presence and absence of ligands might enable its use as a calibrated force probe in translocation measurements and yield information about the mechanical strength of the SecA motor.”

9. *There are multiple places where the text if figures is too small to read on a printed page (at least for an older persons eyes). For instance, I can’t read the subscripts on k in Figure 1D and also couldn’t distinguish the gray curve on top of the orange line. I had to blow it up on my monitor. The data is lovely, don’t make people work so hard to see it. I also couldn’t read the grey text in the legend of Figure 2D.*

We have adjusted the font sizes of labels and/or subscripts in Figures 1C-F and 4D, and the font color in Figure 2D-F to increase legibility. We also changed the color of the curve representing the fits in Figure 1D-F to black. We hope that these changes increase legibility of the figures and labels.

0. *When transforming a rupture force distribution onto a k(F) vs F plot, the experimental data falls onto a portion of that plot (rather than the curve used in the present manuscript). What force range was that and did and how far did the authors need to extrapolate from their data to the regime where they are interpreting the data at 10 pN. To be clear, I am thinking of multitude of loading rates similarly transformed shown in a recent review (Figure 6D, Woodside and Block, Annu Rev Biophysics 2014). I am not asking the authors to take data rather clarify if there experimental data is needed to be extrapolated in the k(F) vs F graph.*

The figure from the Woodside and Block presents unfolding rates from force ramp measurements in an appealing way. The data (from Greenleaf *et a!*. 2008, PMID 18174398) was obtained with a Dudko-type analysis that is in principle similar to our analysis. However, Greenleaf *et a!*. first divided their unfolding forces into discrete bins and obtained an average lifetime for each bin (represented by the data points in the Woodside and Block review Figure 6D). In contrast, we developed here a maximum likelihood approach that omits data binning. The approach avoids the averaging associated with binning. However, it does not yield lifetimes at discrete forces. As such, we cannot generate an analogous plot for our data. The reviewer is correct that we extrapolated the experimental data using the fit result shown in our Figure 4C. We have indicated the range of our experimental data in the revised Figure 4C (horizontal bars).

**Reviewer comments, second version:**

Reviewer #2 (Remarks to the Author):

The authors have greatly improved the manuscript since initial submission. I find their answers to my questions about fitting their model to the translocation data to be very convincing. In fact, their SI discussion of parameter sensitivity analysis (p. 19-20) could serve as a valuable primer to other investigators attempting to fit many-parameter models to data.

I am pleased that the authors have reframed their argument for the existence of two folded states of the apo protein so that it does not depend on Fig. S5, but instead on Fig. 2E/F and the prior work of Thillet et al. There are two refinements to this argument that I think the authors should consider. These points are sufficiently minor that, once they are made, I do not need to review the manuscript again before it can be accepted.

1. The reference to Thillet et al. and the analysis of Fig. 2E/F make clear the existence of competing states in the folded apo protein. It would be useful to the reader to include a sentence explaining why there are not similarly two states in the folded ligand-bound protein. I assume this argument follows from Scheme I of Thillet et al. and the binding affinity of the ligand to the protein.

2. The concentrations of NADPH and MTX used in the measurements should be quoted somewhere; I couldn’t find them.

**Author rebuttal, second version:**

Reviewer #2 (Remarks to the Author):

The authors have greatly improved the manuscript since initial submission. I find their answers to my questions about fitting their model to the translocation data to be very convincing. In fact, their SI discussion of parameter sensitivity analysis (p. 19-20) could serve as a valuable primer to other investigators attempting to fit many-parameter models to data.

We are delighted that the reviewer is convinced by the additional information provided in the revised version and wish to thank the reviewer again for the thorough review and constructive comments that have greatly helped to improve the manuscript.

I am pleased that the authors have reframed their argument for the existence of two folded states of the apo protein so that it does not depend on Fig. S5, but instead on Fig. 2E/F and the prior work of Thillet et al. There are two refinements to this argument that I think the authors should consider. These points are sufficiently minor that, once they are made, I do not need to review the manuscript again before it can be accepted. We agree with the reviewer that this point deserves additional clarification.

1. The reference to Thillet et al. and the analysis of Fig. 2E/F make clear the existence of competing states in the folded apo protein. It would be useful to the reader to include a sentence explaining why there are not similarly two states in the folded ligand-bound protein. I assume this argument follows from Scheme I of Thillet et al. and the binding affinity of the ligand to the protein.

To better explain why the ligand-bound, folded protein adopts only one stable state, we have added the following sentences to the Results section (p. 7 in the revised manuscript):

“Notably, the unfolding force distributions obtained with liganded mDHFR are consistent with a single folded state, in contrast to the observation of multiple states of the apo protein. The ligand concentrations used here are well above the dissociation constants 49, and the enzyme therefore is mostly in the ligand-bound state, which appears to be mechanically very stable.”

2. The concentrations of NADPH and MTX used in the measurements should be quoted somewhere; I couldn’t find them.

We thank the reviewer for pointing out this oversight. The concentrations of MTX and NADPH used in the experiments are now given in the Methods section (p. 12 in the revised manuscript):

“Experiments conducted in the presence of MTX included 10 µM MTX in the buffer. Experiments conducted in the presence of both MTX and NADPH contained 10 µM MTX and 10 mM NADPH in the buffer. Experiments for the apo conditions did not contain either ligand.”

**Reviewer comments, third version:**

**Author rebuttal, third version:**
